# Quantifying intracellular trafficking of silica-coated magnetic nanoparticles in live single cells by site-specific direct stochastic optical reconstruction microscopy

**DOI:** 10.1186/s12951-021-01147-1

**Published:** 2021-11-29

**Authors:** Suresh Kumar Chakkarapani, Tae Hwan Shin, Seungah Lee, Kyung-Soo Park, Gwang Lee, Seong Ho Kang

**Affiliations:** 1 Department of Chemistry, Graduate School, Kyung Hee University, Yongin-si, Gyeonggi-do 17104 Republic of Korea; 2 Department of Physiology, Ajou University School of Medicine, 164, World cup-ro, Yeongtong-gu, Suwon-si, Gyeonggi-do 16499 Republic of Korea; 3 Department of Applied Chemistry and Institute of Natural Sciences, Kyung Hee University, Yongin-si, Gyeonggi-do 17104 Republic of Korea; 4 Nanophotonics Research Center, Korea Institute of Science and Technology, Seoul, 02792 Republic of Korea; 5 Department of Molecular Science and Technology, Ajou University, Suwon-si, Gyeonggi-do 16499 Republic of Korea

**Keywords:** Magnetic nanoparticle, Super-resolution microscopy, Single-particle tracking, Microarray, Live cell analysis

## Abstract

**Background:**

Nanoparticles have been used for biomedical applications, including drug delivery, diagnosis, and imaging based on their unique properties derived from small size and large surface-to-volume ratio. However, concerns regarding unexpected toxicity due to the localization of nanoparticles in the cells are growing. Herein, we quantified the number of cell-internalized nanoparticles and monitored their cellular localization, which are critical factors for biomedical applications of nanoparticles.

**Methods:**

This study investigates the intracellular trafficking of silica-coated magnetic nanoparticles containing rhodamine B isothiocyanate dye [MNPs@SiO_2_(RITC)] in various live single cells, such as HEK293, NIH3T3, and RAW 264.7 cells, using site-specific direct stochastic optical reconstruction microscopy (*d*STORM). The time-dependent subdiffraction-limit spatial resolution of the *d*STORM method allowed intracellular site-specific quantification and tracking of MNPs@SiO_2_(RITC).

**Results:**

The MNPs@SiO_2_(RITC) were observed to be highly internalized in RAW 264.7 cells, compared to the HEK293 and NIH3T3 cells undergoing single-particle analysis. In addition, MNPs@SiO_2_(RITC) were internalized within the nuclei of RAW 264.7 and HEK293 cells but were not detected in the nuclei of NIH3T3 cells. Moreover, because of the treatment of the MNPs@SiO_2_(RITC), more micronuclei were detected in RAW 264.7 cells than in other cells.

**Conclusion:**

The sensitive and quantitative evaluations of MNPs@SiO_2_(RITC) at specific sites in three different cells using a combination of *d*STORM, transcriptomics, and molecular biology were performed. These findings highlight the quantitative differences in the uptake efficiency of MNPs@SiO_2_(RITC) and ultra-sensitivity, varying according to the cell types as ascertained by subdiffraction-limit super-resolution microscopy.

**Graphical Abstract:**

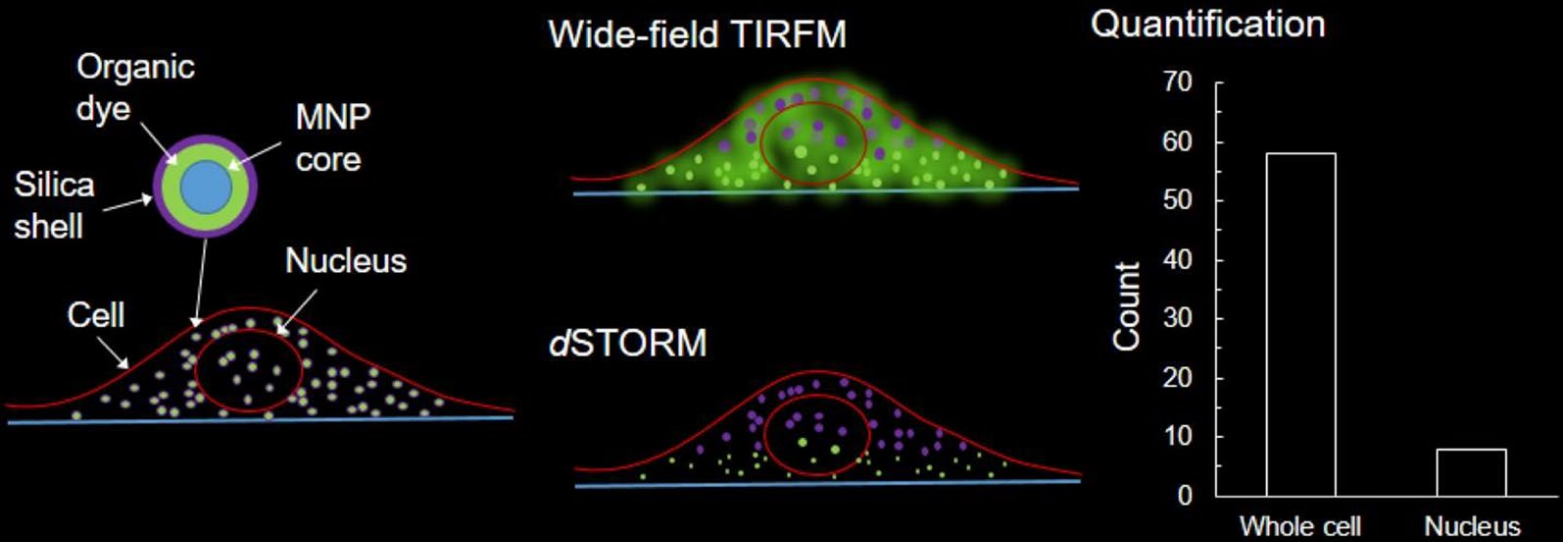

**Supplementary Information:**

The online version contains supplementary material available at 10.1186/s12951-021-01147-1.

## Background

Engineered magnetic nanoparticles (MNPs), because of their inert chemical composition and ease of modification, serve as important nanomedicine probes for the potent delivery of genes and drugs to cells [1]. MNPs and MNPs coated with biocompatible compounds are widely studied for uses in drug delivery, as contrast agents in magnetic resonance imaging (MRI), and in cell labeling [1–3]. Among various types of MNPs, silica-coated magnetic nanoparticles containing rhodamine B isothiocyanate dye [MNPs@SiO_2_(RITC)] possess drug-delivering capabilities in which rhodamine B isothiocyanate (RITC) and MNP provide versatile ways for monitoring using fluorescence microscopy and MRI [4, 5]. The MNPs*@*SiO_2_(RITC) have novel applications as drug and gene nanocarriers, MRI contrast agents, and in antibody engineering. In particular, highly toxic cobalt ferrite nanoparticles (NPs) could be widely applied for surface conjugation studies of drugs or antibodies rather than nanomedical encapsulation for drug delivery study [6, 7]. Precise quantitative estimation of cellular NP uptake and the consequent internalization of NPs at the intracellular sites is needed to support the use of MNPs for high-profile medical applications. The quantitative estimation of such drug carriers at intracellular sites is essential for determining the pharmacokinetics, that is, the molecules delivered by native carriers and their respective cellular effects.

Quantitative assessment of the in vitro cell effects of nanodrug carriers on macrophage cells holds great interest for diagnostic and therapeutic applications because these effects are crucial to innate immunity. The macrophage cell plays an important role in the initiation, maintenance, and resolution of inflammation [8]. Because of its pivotal role, the macrophage cell is an important target for drug carriers such as MNPs*@*SiO_2_(RITC) in the treatment of a large number of diseases. Macrophage cells are reported to show efficient uptake of macro-size to nano-size particles by the differentiation process corresponding to various cell receptor expressions [9, 10]. Earlier studies suggested that the cell uptake of MNPs*@*SiO_2_(RITC) was mediated through an energy-dependent pathway [11]. However, a complete understanding of the internalization of MNPs*@*SiO_2_(RITC) at a specific site of a cell and the related effects at lower concentrations remain unknown because we lack the appropriate monitoring tools. An ideal imaging platform for addressing this issue will offer three features—the capacity to produce super-resolution images for intracellular site-specific analysis, potential for quantitative estimates of the intracellular MNPs*@*SiO_2_(RITC) in single-cell nanostructures, and ability to dissect the images to distinguish individual MNPs*@*SiO_2_(RITC) in the densely labeled region in live single cells.

Fluorescence, fluorescence-free, and electron microscopy are the imaging platforms that possess the qualities needed for generating intracellular site-specific quantitative NPs images. Electron microscopy provides high throughput imaging with an optimum spatial resolution for quantifying the NPs at the level of single cells [12]. However, obtaining high-quality images requires laborious sample preparation and rigorous fixation steps [13]. In addition, the high-resolution imaging of densely labeled cells is still a challenge in the quantitative estimation of NPs using an electron microscope [13]. In contrast, fluorescence-free microscopic techniques, such as dark field [14] and differential interference contrast (DIC) [15], were reported to be effective tools for imaging NPs at the single-cell level. These techniques offer the advantage of identifying single NPs against proximate neighbors within the diffraction limitation [16, 17]. Recent developments in enhanced dark-field and integrated light-sheet techniques show the possibilities of distinguishing individual intracellular NPs within the diffraction-limited resolution of optical microscopy [18–20]. However, these fluorescence-free microscopes require plasmonic metal NPs, such as silver, gold, and copper, for their imaging probes. Therefore, fluorescence microscopy is the appropriate tool for analyzing internalization of MNPs*@*SiO_2_(RITC) in single cells. Nevertheless, the major drawback of fluorescence microscopy remains its diffraction-limited resolution [21]. According to the limitation, conventional microscopy such as wide-field total internal reflection fluorescence (TIRF) could not resolve the individual NPs that reside close to one another. Obtaining quantitative information about the NPs in the densely internalized cellular regions remains difficult. Therefore, visualizing individual NPs requires fluorescence-based super-resolution microscopy [22, 23]. Several contemporary techniques can resolve structures below the diffraction limit through different approaches. In practical terms, single-molecule localization microscopy (photoactivated localization microscopy, PALM; direct stochastic optical reconstruction microscopy, *d*STORM) can achieve a higher resolving power than stimulated-emission depletion (STED) and structured-illumination microscopy (SIM), reaching ~ 20–50 nm lateral and ~ 10–70 nm axial resolutions [24, 25]. In *d*STORM experiments, commonly used fluorophores, which are used in conventional epifluorescence microscopy, can be combined with specialized buffers and lasers to induce photo-switching. In addition, as *d*STORM does not require a specialized fluorescent probe, lower time and effort is required for optimizing experimental conditions, making it an accessible technique for researchers. The RITC in MNPs*@*SiO_2_(RITC) provides the opportunity for performing stochastic photo-switching to visualize NPs within the bounds of the subdiffraction-limit super-resolution. Therefore, we employed a super-resolution reconstruction platform based on *d*STORM [21, 26–29].

Herein, we consider the biomedical importance of MNPs*@*SiO_2_(RITC), focusing our study on using *d*STORM to develop a better understanding of the internalization of the NPs in three different cell lines. NPs containing fluorescent proteins (i.e., mCherry and green fluorescent protein) and naturally occurring autofluorescent substances reportedly have non-specific adsorption potential [30–33], while non-specific adsorption of fluorescent dyes has not yet been reported for silica or cobalt ferrite. MNPs*@*SiO_2_(RITC) was quantified at the monocellular level with a subdiffraction-limit resolution for the first time at the lowest possible concentration. Although our research focused on the internalization of MNPs*@*SiO_2_(RITC) in macrophage cell lines, a comparison was made with HEK293 and NIH3T3 cell lines to form a better understanding of the sensitivity of internalization. In addition, analyzing the kinetics and dynamics of the internalization of MNPs*@*SiO_2_(RITC) provides good insight into the mechanisms shaping the intracellular uptake process.

## Methods

### Coverslip preparation for *d*STORM imaging

No. 1 (thickness, 0.13–0.16 mm) Corning glass coverslips with dimensions 22 × 22 mm (Paul Marienfeld GmbH & Co. KG, Lauda-Konigshofen, Germany) were washed with deionized water (Human Power 1 + , Human Corporation, Seoul, Korea) and spectroscopic-grade ethanol (Duksan Pure Chemical Co. Ltd., Ansan, Korea), followed again by washing with ultrapure water and spectroscopic-grade methanol (Duksan) before air-drying under UV light for 30 min. A mixture of 10 µg/mL poly-_L_-lysine (PLL, Sigma-Aldrich, St. Louis, MO, USA) and 20 µg/mL laminin (Sigma-Aldrich, St. Louis, MO, USA) was coated on the precleaned coverslips and incubated for 3 h at room temperature.

### Cell culture and labeling with MNPs*@*SiO_2_(RITC)

RAW 264.7, HEK293, and NIH3T3 cells were maintained in T25 plastic tissue culture dishes containing DMEM (Gibco, 12800-058, Grand Island, NY, USA) with 1% antibiotic–antimycotic agent (Gibco, 15240-062, Grand Island, NY, USA) and 10% fetal bovine serum (FBS; Gibco, 26140-079, Grand Island, NY, USA) at 37 °C in a humidified 5% CO_2_ incubator. Every two days, HEK293 and NIH3T3 cells were subcultured by trypsinization, and RAW 264.7 cells were subcultured with a cell lifter. The cells were plated on PLL/laminin-coated glass coverslips and incubated overnight in a humidified incubator at 37 °C. MNPs@SiO_2_(RITC) consisted of a ~ 9-nm cobalt ferrite core (CoFe_2_O_3_) and a RITC-incorporating silica shell [3] were purchased from Biterials (Seoul, South Korea). The size of MNPs@SiO_2_(RITC) is 50 nm, and *zeta* potential is between − 40 and − 30 mV [3, 34]. Rhodamine B containing SiO_2_ NPs (10-nm, 50-nm, and 200-nm size) were purchased from CD Bioparticles (Shirley, NY, USA). We confirmed the structure of the MNPs@SiO_2_(RITC) with X-ray diffraction (XRD) using a High-Power X-Ray Diffractometer (Ultima III, Rigaku, Japan), which showed specific patterns of CoFe_2_O_4_ and amorphous silica beads (data not shown). The cells were washed three times with phosphate-buffered saline (PBS) and labeled with the desired concentration of MNPs*@*SiO_2_(RITC) and SiO_2_ NPs in FBS-free DMEM. Before data acquisition, excess NPs were squeezed out and the cells were washed three times with PBS. The coverslips with NP-labeled cells were placed on the dove prism and *d*STORM solution [10 mM *β*-mercaptoethylamine (MEA), 0.5 mg/mL glucose oxidase, 40 μg/mL catalase, 10% glucose (Sigma-Aldrich, St. Louis, MO, USA)] was added on top of the cells, which were incubated for 15 min prior to imaging.

Primary liver cells were isolated from 8-week-old male C57BL/6 mice (Doo Yeol Biotech, Seoul, Korea), as previously described [35–37]. Experimental protocols were approved by the Laboratory Animal Research Center of Ajou University Medical Center. Briefly, mice were anesthetized using isoflurane prior to the abdominal operation. The liver was perfused with perfusion buffer (10 mM HEPES, 140 mM NaCl, 6 mM KCl, and 2 mM EGTA, pH 7.4) for 5 min at a flow rate of 7 mL/min. The liver was then re-perfused with collagenase buffer [0.48 mM HEPES, 66.7 mM NaCl, 6.7 mM KCl, 0.48 mM CaCl_2_, and 0.8 mg/mL collagenase type IV (Sigma-Aldrich, St. Louis, MO, USA), pH 7.4] and extracted from the mouse body. The extracted liver was homogenized, and primary hepatocytes were isolated by centrifugation at 50×*g* for 5 min and layered using a 40% Percoll cushion by centrifugation at 300×*g* for 7 min. The isolated hepatocytes were incubated on plates and cover glasses coated with collagen type I (Sigma-Aldrich, St. Louis, MO, USA) in growth medium [Medium 199 (Thermo Fisher Scientific, Sunnyvale, CA, USA) with 10% FBS and 1% antibiotic–antimycotic agent]. Liver non-parenchymal cells fraction, containing Kupffer cells, liver sinusoidal endothelial cells, and stellate cells, was collected from the homogenized liver by layering with 25% and 50% Percoll gradient using centrifugation at 1800×*g* for 30 min. The layer, containing Kupffer cells and liver sinusoidal endothelial cells, was separated into Kupffer cells and liver sinusoidal endothelial cells by selective adhesion [37]. The layer was transferred to a culture plate and cover glass with RPMI 1640 media (Thermo Fisher Scientific, Sunnyvale, CA, USA) containing 10% FBS and 1% antibiotic–antimycotic agent and incubated for 8 min at room temperature to ensure adhesion of Kupffer cells onto the plate and cover glass. Non-adhered cells (liver sinusoidal endothelial cells) were collected and transferred to a collagen type I-coated plate and cover glass containing DMEM (Gibco, Grand Island, NY, USA) with 10% FBS and 1% antibiotic–antimycotic agent. The three types of cells were incubated at 37 °C in a humidified 5% CO_2_ incubator. The isolated mouse primary hepatocytes, Kupffer cells and liver sinusoidal endothelial cells were characterized based on morphological analysis and specific marker protein expression: albumin for hepatocytes, F4/80 [encoded in adhesion G protein coupled receptor E1 (*ADGRE1*) gene] for Kupffer cells, platelet endothelial cell adhesion molecule [*PECAM-1*, also known as cluster of differentiation 31 (*CD31*)] for liver sinusoidal endothelial cells (Additional file 1: Fig. S1).

### *d*STORM setup and data acquisition

A lab-made dove prism-type TIRF setup, resembling the apparatus previously used in our group, but with some modifications, was employed (Additional file 1: Fig. S2) [38]. The setup consisted of an Olympus BX53 upright microscope (Olympus Optical Co., Ltd., Tokyo, Japan) equipped with a × 100-oil type objective lens with numerical aperture 1.4 (Olympus, UPlanSApo) and a DIC slider with Nomarski prism (Olympus, U-DICT). The dove prism with the sample was placed on the prism holder. Light sources from 532-nm-(Changchun New Industries Optoelectronics Tech. CO., Ltd., Jilin, China) and 405-nm-wavelength lasers (COMPACT-30G-405, World Star Tech., Toronto, ON, Canada) were directed to the prism with the help of mirrors to obtain total internal reflection and to produce an evanescent field layer on the interface. An electron-multiplying cooled charge-coupled device camera (EM-CCD, 512 × 512 pixels imaging array, iXon Ultra, Andor, Belfast, Ireland), along with a 575/15 nm band-pass filter (Semrock, Rochester, NY, USA) placed on the optical path of the microscope, was used to acquire the fluorescence images. MetaMorph 7.8.12.0 software (Molecular Devices, LLC, Sunnyvale, CA, USA) was used to control the image acquisition, shutter speed, and exposure time of the camera.

### dSTORM image analysis

Acquired images were analyzed using a ThunderStorm plugin in ImageJ (http://rsbweb.nih.gov/ij/) software. For superlocalization, the centroid of the fluorescence spot was fitted to the two-dimensional (2D) Gaussian function [39]:

$$f\left(x, y,{z}_{o},A,{x}_{o},{y}_{o},{s}_{x},{s}_{y}\right)={z}_{o}+A exp\left[-\frac{1}{2}\left[{\left(\frac{(x-{x}_{o}}{{s}_{x}}\right)}^{2}-{\left(\frac{y-{y}_{o}}{{s}_{y}}\right)}^{2}\right]\right]$$

where *s*_*x*_ and *s*_*y*_ are the standard deviations along directions *x* and *y*, respectively, *x*_*o*_ and *y*_*o*_ are coordinates of the centers, *z*_*o*_ is a constant from background noise, and *A* is the amplitude. Localization precision *σ* [40] was calculated with.

$${\varvec{\sigma}}=\sqrt{\left(\frac{{s}^{2}}{N}+\frac{{a}^{2}}{12N}+\frac{8\pi {s}^{4}{b}^{2}}{{a}^{2}{N}^{2}}\right),}$$

where *N* is the number of collected photons, *a* is the pixel size of the detector, *s* is the standard deviation of the point-spread function, and *b* is the background noise of the detector. From the reconstructed *d*STORM images, the individual NPs were identified in large fields of view, and their properties were assessed with a fitting procedure. By the means of aggregated data set and mean value of the fitted spots, individual NPs were investigated and quantified. The *d*STORM images of live cells were drift-corrected using the bright spots on the coverslips as reference markers. The bright spots on coverslips were used as fiducial markers. The image contained live cell and fiducial marker was acquired. The acquired images were reconstructed by ThunderSTORM to obtain the NP distribution in cells. After superlocalization, the image of fiducial marker was loaded and drift correction was carried out using ThunderSTORM automatically (Additional file 1: Fig. S3) [41]. The NP number in cells was counted by TrackMate, a plugin of ImageJ (NIH, Bethesda, Maryland, USA, https://imagej.nih.gov/ij/).

### Cell viability assay

For the cell viability assay, an MTS [(3-(4,5-dimethylthiazol-2-yl)-5-(3-carboxymethoxyphenyl)-2-(4-sulfophenyl)-2H-tetrazolium)] assay was performed as described in a previous study [21, 42, 43], using a CellTiter 96-cell proliferation assay kit (Promega Corporation, Madison, WI, USA) according to the manufacturer’s instructions. In this step, 2 × 10^4^ cells were seeded on a 96-well assay plate and treated with MNPs@SiO_2_(RITC) and SiO_2_ NPs for 12 h. MTS solution was added to each well of the 96-well assay plate containing treated cells in 100 µL of culture medium. The assay plate was then incubated for 1 h under 5% CO_2_ at 37 °C. The amount of soluble formazan produced by cellular reduction was directly measured with a plate reader at a wavelength of 490 nm. The values were normalized relative to the protein optical density value for each corresponding group.

### Microarray analysis

Gene expression profiles were analyzed as described in a previous study [44–47]. In this step, changes in gene expressions of MNPs@SiO_2_(RITC)-treated cells (0.1 mg/mL, 1.0 mg/mL) were examined using a Human U133 Plus 2.0 50 K Affymetrix system (ISTECH Corp., Korea), which contains 54,675 probes. Biological pathways were enriched using the web-based bioinformatics software Ingenuity Pathway Analysis (IPA; Qiagen, Valencia, CA, USA). A threefold change in gene expression was used as a cut-off to generate data sets of significantly changed genes.

### Real-time polymerase chain reaction (PCR)

The expressions of DNA repair-related genes were determined by using a real-time PCR kit, based on RealMOD™ SYBR Green (Intron, Seong-Nam, Korea), that employed Rotor Gene-Q (Qiagen, Valencia, CA) with gene-specific primer pairs (Additional file 1: Table S1). The PCR reactions were carried out at 95 °C for 5 min followed by 50 cycles of 95 °C for 5 s and 60 °C for 30 s, according to the manufacturer’s protocol. The threshold/quantification cycle (Ct/Cq) value was determined at the point in which fluorescence was detected statistically above the background, and PCR products were analyzed by generating a melting curve, constructed by Rotor-Gene 1.7 software (Qiagen, Valencia, CA). PCR was run in independent triplicates. The relative quantification of these gene expressions was calculated by the 2^−ddCt^ method.

### Quantification of micronuclei

Cells were cultured in a 24-well plate (SPL, Suwon, Korea) and treated with MNPs@SiO_2_(RITC) for 12 h. The cells were then stained with 10 µg/mL of Hoechst33342 (Invitrogen, Carlsbad, CA, USA) in PBS for 10 min at room temperature and washed three times with PBS. Fluorescent images were acquired by Axio Vert 200 M fluorescence microscopy (Zeiss, Jena, Germany) at the Three-Dimensional Immune System Imaging Core Facility of Ajou University. The excitation wavelengths for Hoechst33342 and MNPs@SiO_2_(RITC) were 405 and 530 nm, respectively. Micronuclei were counted in five to seven randomly chosen areas on each well. More than 200 cells per experimental group were counted using ImageJ. The statistical analysis was done by assessing the results by one-way analysis of variance (ANOVA) with Bonferroni’s multiple-comparison tests using IBM-SPSS software (IBM Corp., USA). In all analyses, *p* < 0.05 was taken to indicate statistical significance.

### Inductively coupled plasma/quadrupole mass spectrometer (ICP-QMS) and atomic absorption spectrometer (AAS) analysis

An inductively coupled plasma/quadrupole mass spectrometer (PerkinElmer, MA, USA) was used for the quantitative estimation of Co. The analysis was done with a 1600 W RF power supply, 18.0 L/min coolant gas-flow rate, 1.30 L/min auxiliary gas-flow rate, and 0.98 L/min nebulizer gas-flow rate. For quantitative estimation of Fe, an atomic absorption spectrometer (Thermo Fischer, MA, USA) was used along with a flame atomizer system and an air-acetylene fuel with a 1.0 L/min gas flow at a 248.3 nm wavelength.

The nucleus fraction was prepared with Nuclei EZ™ prep kit (Sigma-Aldrich, St. Louis, MO, USA). Isolation procedures were performed according to the manufacturer’s instructions. In this step, 3 × 10^6^ cells were washed with ice-cold PBS and lysed with Nuclei EZ lysis buffer. The cells were harvested with a scraper and incubated for 5 min on ice. The lysed fractions were pelleted by centrifugation at 500 g for 5 min at 4 °C. The pellets were washed with Nuclei EZ lysis buffer and vortexed. Final nuclei fractions were collected by centrifugation at 500*g* for 5 min at 4 °C and resuspended with a Nuclei EZ storage buffer.

## Results

### Localization of MNPs***@***SiO_2_(RITC) with ***d***STORM

The rates of fluorescence in the off and on states were observed with 532-nm- and 405-nm-wavelength lasers to study the photo-switching conditions of RITC in MNPs*@*SiO_2_(RITC). The 50 nm MNPs*@*SiO_2_(RITC) was placed on PLL and laminin-coated cover glasses and irradiated with a 532 nm laser in the presence of a 100 mM MEA buffer. A linear dependence of first-order rate constants was observed in relation to the excitation wavelength intensities. The rate for the off state (Additional file 1: Fig. S4a) was obtained by the singlet state excitation with a 532 nm laser. The rate for the on the state (Additional file 1: Fig. S4b) was obtained by the simultaneous illumination of a 405 nm laser. Both rate constants showed a linear dependence on the laser power intensities. The sample was irradiated with a 532-nm laser and then with a 405-nm activation laser. This process of illumination assured that a constant number of fluorophores occupied the fluorescent state at any given time (Additional file 1: Fig. S4c).

HEK293, NIH3T3, and RAW 264.7 cells were incubated with three different concentrations at different incubation times (Additional file 1: Figs. S5, S6) to optimize their concentrations for the analysis of MNPs*@*SiO_2_(RITC) with *d*STORM imaging. Because NPs are not reagents to which specific antibodies are bound or stained for specific organelles in cells, the optimization of the concentration of NPs was determined through intuitive characterization rather than localization density [48]. At a higher concentration (0.1 μg/mL), RAW 264.7 cells demonstrated the uptake of numerous NPs, making the task of distinguishing individual NPs difficult in practice. At a lower concentration (0.003 μg/mL), however, NIH3T3 cells displayed the uptake of few NPs, even after undergoing incubation for longer durations. Thus, a 0.01 μg/mL concentration of MNPs*@*SiO_2_(RITC) was considered optimum for performing a *d*STORM quantitative analysis. The HEK293 cells were incubated with 0.01 µg/mL of MNPs*@*SiO_2_(RITC), and the sample was irradiated simultaneously with 532 nm and 405 nm lasers. The movie M1 shows the photo-switching (Additional file 2: Movie S1) of MNPs*@*SiO_2_(RITC) in the HEK293 cells. The photo-switching cycles were repeated over 10,000 frames, and the resultant frames were reconstructed into a single image. The cell morphology was maintained as per normal cell morphology in culture conditions until imaging and data acquisition through *d*STORM analysis.

**Fig. 1:**
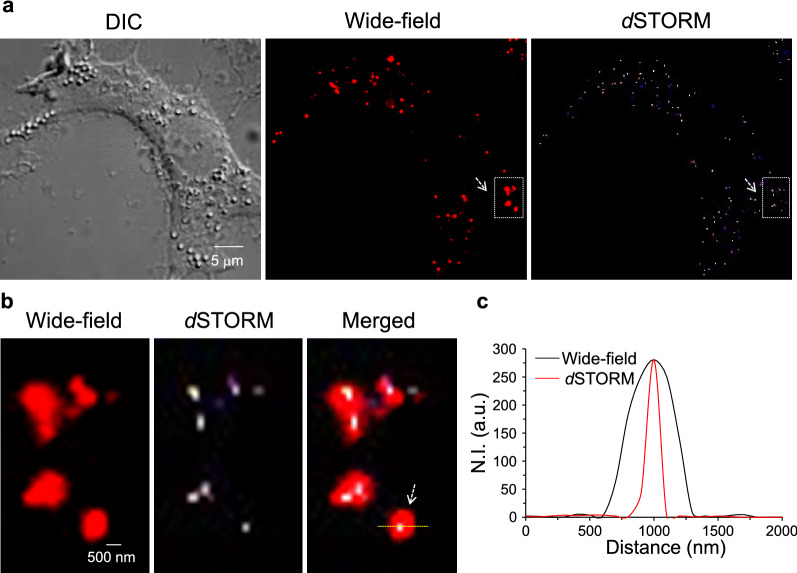
Intracellular localization of single NPs. **a** DIC, wide-field, and *d*STORM images of an HEK293 cell internalized with 50-nm-sized MNPs*@*SiO_2_(RITC). **b** The insets of the wide-field and *d*STORM images of (**a**). The merged image layers the wide-field and *d*STORM images to show the localization of NPs by super-resolution imaging and line-scan profiles for the normalized intensity (N.I.) of wide-field and *d*STORM images of a single NP in (**c**)

Figure 1a shows the DIC and wide-field TIRF images of the HEK293 cell. The noticeable exceptions occurred where the NP images in wide-field TIRF conditions were under the diffraction limitation; the resolution of the image did not meet the accepted standards. The *d*STORM image shows the reconstructed super-resolution image of the same cell. A portion of the image is magnified to show the resolution difference between the wide-field TIRF and *d*STORM images (Fig. 1b). Under super-resolution conditions, individual NPs were distinguished and helped to quantify the intercellular NPs. The image created by merging the wide-field and *d*STORM images (Fig. 1b) shows the localization of individual NPs that cannot be resolved by conventional wide-field fluorescence microscopy. The line-scan profile plot (Fig. 1c) of a single NP showed a six-fold difference in higher resolution in comparison to that of a super-resolution image. The NPs residing as close to one another were not resolved by conventional TIRF wide-field microscopy. Thus, with photo-switching events and localization clusters of the centroid, the individual NPs as close as 80 nm were resolved (Additional file 1: Fig. S7).

### MNPs*@*SiO_2_(RITC) internalization in three different cell lines

**Fig. 2:**
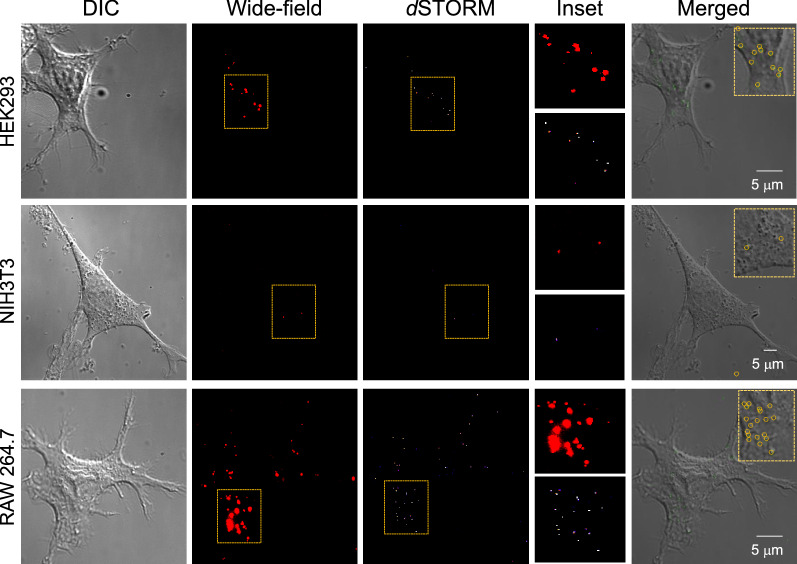
Super-resolution images of internalized MNPs*@*SiO_2_(RITC) for three different cells. DIC, wide-field, *d*STORM, insets of wide-field and *d*STORM, and merged DIC–*d*STORM images of HEK293, NIH3T3, and RAW 264.7 cells, respectively. Images were acquired after incubation of 50 nm MNPs*@*SiO_2_(RITC) for 1 h with each cell. The merged images of DIC and *d*STORM support site-specific analysis of the internalized NPs

The NP internalization was performed with PBS and serum-free growth media. The quantification of NPs with both was observed to be the same as with RAW 264.7 cells (Additional file 1: Fig. S8). Therefore, to characterize the NP uptake of RAW 264.7 cells alongside HEK293 and NIH3T3 cells, we incubated cells with MNPs*@*SiO_2_(RITC) in serum-free growth media. The growth media avoided cellular damage at longer incubation times. The experiment was performed with all three types of cells under the same incubation conditions at 37 °C for energy-dependent uptake. After incubation for 1 h, all three cell samples were observed to have internalized NPs (Fig. 2).

*d*STORM imaging was performed under the same conditions for all three cells, and the NPs were localized in each cell. Cells showed different quantities of internalized NPs with RAW 264.7 cells emerging as the one with the most uptake among the three. More NPs were internalized with the RAW 264.7 cells than with the other cells at the same concentration of NP treatment. NIH3T3 showed comparatively low uptake efficiency, where only two to three NPs were internalized after an incubation of 1 h. These results were correlated with the previously reported results that macrophage cells exhibited higher efficiency than other cell lines [49, 50]. Quantification of the NPs at the specific site of the cells was made by merging the DIC images with the *d*STORM images of the cells. The merged image showed the internalized NPs in the specific site of the cells, thus helping with the site-specific analysis of internalized NPs.

### Assessment of toxicity in MNPs*@*SiO_2_(RITC) treated cells

**Fig. 3:**
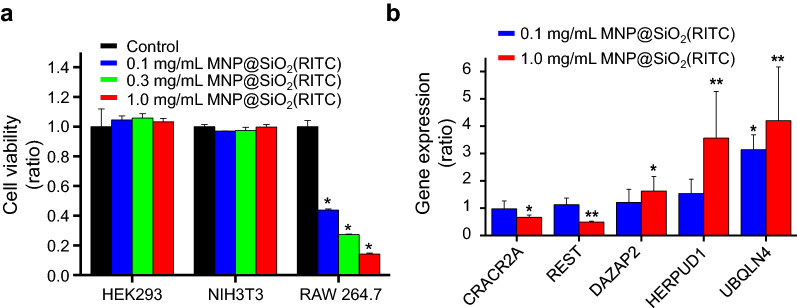
Cell viability and DNA-repair-related genes’ expression in MNPs*@*SiO_2_(RITC) internalized cells. **a** MTS assay of MNPs@SiO_2_(RITC)-treated HEK293, NIH3T3, and RAW 264.7 cells. Cells were treated with 0.1 mg/mL, 0.3 mg/mL, and 1.0 mg/mL MNPs@SiO_2_(RITC) for 12 h, followed by cell viability assessment using an MTS assay. **b** Quantitative analysis of DNA repair-related genes using quantitative real-time PCR (qPCR). HEK293 cells were treated with 0.1 mg/mL and 1.0 mg/mL of MNPs@SiO_2_(RITC) for 12 h. qPCR was performed using specific primers for target genes *CRACR2A*, *REST*, *DAZAP2*, *HERPUD1*, and *UBQLN4*. Gene expression levels of the target genes were normalized relative to the corresponding means in non-treated controls. *GAPDH* was used as an internal control in qPCR. Here, *p < 0.05 and **p < 0.01 in one-way ANOVA compared to the control were considered significantly different

We performed an MTS assay with a conventional cell, labeling MNPs@SiO_2_(RITC) concentration of 0.1 to 1.0 mg/mL (Fig. 3a). In this case, we found significant cell death only in RAW 264.7 cells in the MNPs@SiO_2_(RITC) treatment in a dose-dependent manner. In contrast, we did not find any toxicity in cells at concentrations of 0.1 to 1.0 µg/mL of MNPs@SiO_2_(RITC) (Additional file 1: Fig. S9). In addition, gene expression profiles in MNPs@SiO_2_(RITC)-treated HEK293 cells were significantly changed in 69 DNA repair-related genes in a dose-dependent manner when using IPA (Additional file 1: Figs. S10, S11, Table S2). The changed expression levels of calcium release activated channel regulator 2A (*CRACR2A*), RE1-silencing transcription factor (*REST*), DAZ associated protein 2 (*DAZAP2*), homocysteine-inducible, endoplasmic reticulum stress-inducible, ubiquitin-like domain member 1 (*HERPUD1*), and ubiquilin 4 (*UBQLN4*) were validated using quantitative PCR (Fig. 3b).

**Fig. 4:**
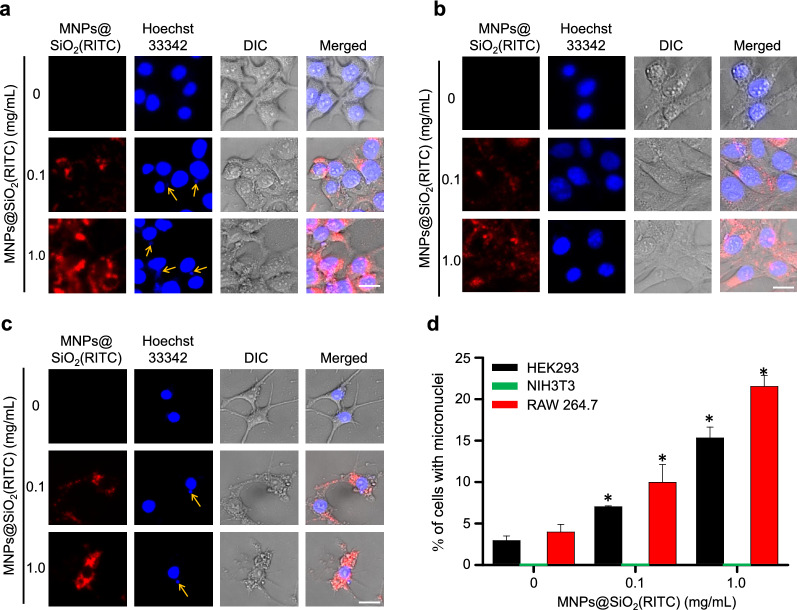
Micronuclei formation in MNPs@SiO_2_(RITC)-treated cells. Optical and fluorescence microscopic appearance of MNPs@SiO_2_(RITC)-treated **a** HEK293, **b** NIH3T3, and **c** RAW 264.7 cells. Here, red and blue indicate MNPs@SiO_2_(RITC) and Hoechst 3334﻿2, respectively. The scale bars in the lower-right corners are 20 μm. The yellow arrows indicate the micronuclei in each cell. **d** The frequencies of micronuclei-containing cells. More than 200 cells per experimental group were counted using ImageJ. Micronuclei were not detected in NIH3T3 (Green). Data represent mean ± S.D. related to the control of three independent experiments. *p < 0.05 in one-way ANOVA compared to each control group was considered significantly different

Micronuclei provide one indicator of genotoxicity and are produced when double-strand breaks occur in a nucleus and replication is accompanied by a nuclear envelope burst [51]. Micronuclei were detected in MNPs@SiO_2_(RITC)-treated HEK293 and RAW 264.7 cells rather than in NIH3T3 cells, and the frequency increased in a dose-dependent manner, showing a more pronounced increase in RAW 264.7 cells (Fig. 4). These results suggested that cell-internalized MNPs@SiO_2_(RITC) may interact with chromosomes in nuclei and induce genotoxicity in the cells.

### Site-specific quantification of cell-internalized MNPs*@*SiO_2_(RITC)

**Fig. 5:**
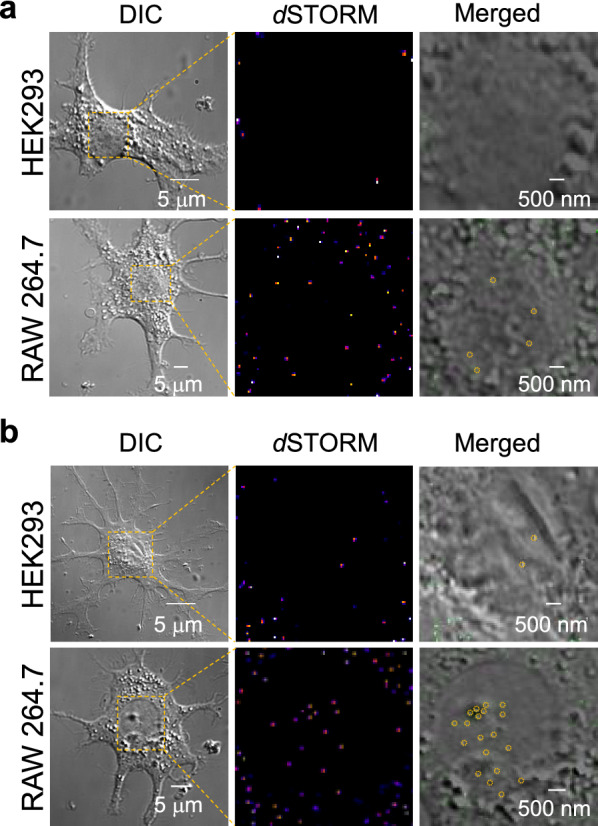
Intranuclear images of internalized MNPs*@*SiO_2_(RITC). DIC, *d*STORM, and DIC merged *d*STORM images of the internalized nanoparticles in HEK293 and RAW 264.7 cells after incubations of **a** 5 h and **b** 12 h. NPs circled with dotted yellow lines indicate that they were internalized in the nuclear regions of the cells

The advantage of using *d*STORM as the detecting tool arises from the possibilities it offers for performing nanostructure analysis of cells. In an application of the *d*STORM technique, the NPs internalized within the nuclear regions of RAW 264.7 cells were observed (Fig. 5a) after a 5 h incubation with NPs. After 12 h of incubation, NPs were internalized in the nuclear region of both RAW 264.7 and HEK293 cells (Fig. 5b). For the estimation of time-dependent internalized MNPs*@*SiO_2_(RITC), three cells were incubated with NPs for different time intervals (Additional file 1: Figs. S12–S14); The NPs were counted manually as individual NPs were resolved by *d*STORM imaging.

**Fig. 6:**
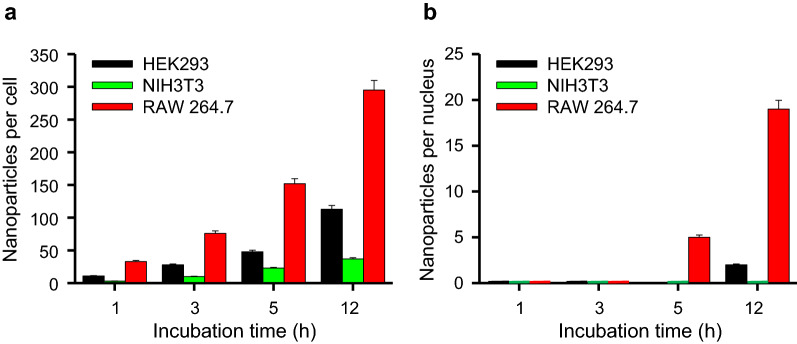
Intracellular quantification of nanoparticles. MNPs*@*SiO_2_(RITC) quantification in HEK293, NIH3T3, and RAW 264.7 cells in **a** the whole intracellular region and **b** the intranuclear region of the cells. Micronuclei were not detected in NIH3T3 (Green). Error bars represent the mean ± S.D. related to the control of three independent experiments

Figure 6 shows the number of NPs internalized in each cell as a whole along with the number of NPs internalized within the nuclear regions of the cells. RAW 264.7 cells showed higher measures of quantitative uptake of NPs compared to HEK293 cells. The NIH3T3 cells showed no NPs in the nuclear regions. In our study, we observed that 50 nm MNPs*@*SiO_2_(RITC) were internalized within the nuclei of both RAW 264.7 and HEK293 cells. Even though a large number of NPs seemed to be located in the cytoplasm and accumulated around the nuclei, forming nuclear indentations, a small fraction of NPs entered the nuclei (Fig. 6b). Thus, the number of NPs internalized in the nuclei was low in comparison with the total number of NPs internalized in the whole region of the cells. The internalization of NPs in the nuclear region was confirmed using ICP-QMS analysis (Additional file 1: Table S3). The analysis used 0.01 µg/mL of MNPs*@*SiO_2_(RITC), which was the same concentration used for *d*STORM imaging. ICP-QMS could not detect the NPs as a whole within the cells because of sensitivity limitations. However, the results with quantification of NPs in the nuclear region showed that NPs were internalized more in the nuclei of RAW 264.7 cells than in those of NIH3T3 cells; this conclusion matches the results obtained from *d*STORM analysis. Moreover, to observe this intranuclear internalization, we performed 3D DIC combined TIRF wide-field imaging for the RAW 264.7 cells (Additional file 3: Movie S2). The movie file confirmed that the NPs were internalized inside the nuclei of the cells.

### Real-time dynamics of intranuclear MNPs*@*SiO_2_(RITC)

**Fig. 7:**
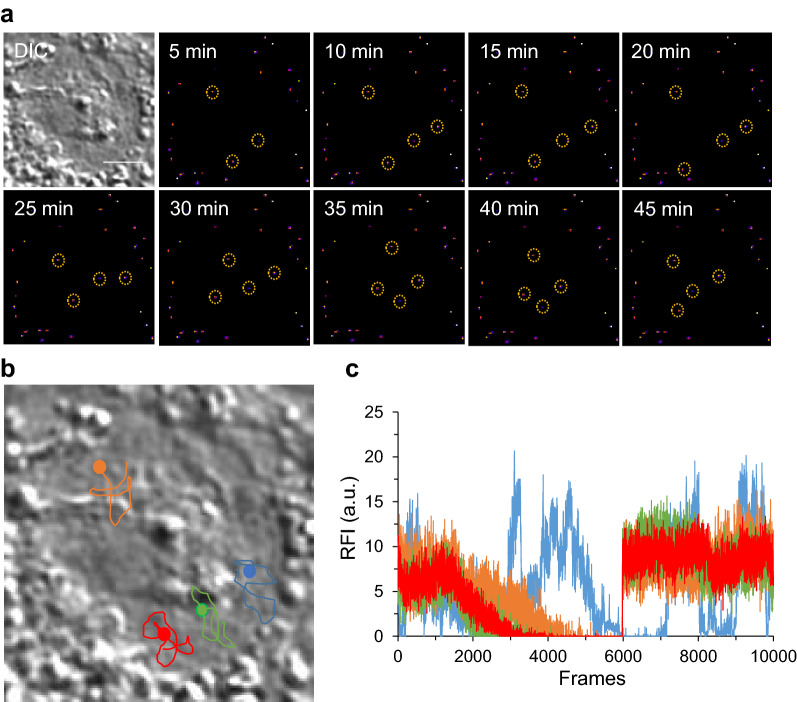
Real-time dynamic images of intranuclear NPs in RAW 264.7 cells. **a** Time-lapse image of individual NPs internalized within the nuclei of the cells. **b** Traces of individual NPs in the nuclei of the cells. **c** Time-lapse fluorescence intensity of individual NPs. Here, RFI stands for relative fluorescence intensity. Scale bar = 5 µm

We tracked NPs in RAW 264.7 cells after 5 h of incubation with 50 nm MNPs*@*SiO_2_(RITC) to obtain visualizations of NP interactions with cell nuclei (Additional file 4: Movie S3). The NPs accumulating around the nuclei did not show any type of movement as they received nuclear indentations (Fig. 7). However, as the NPs entered the cell nuclei, the nuclei ruptured under the influence of protein aggregations. Four NPs (*S*/*N* = 18) were observed within the nuclei and the tracks of the NPs were traced (Fig. 7b). The fluorescence intensities of four NPs were monitored with each frame. The intensity of the NPs fell in the middle of the imaging period, signifying the diffusion of the NPs within the nuclei, however; the intensity increased with time and then fell into a steady state. Thus, the NPs that showed similar intensities and fluctuations in intensity signified the same physical state in the interaction of the NPs with the nuclei of the cells. The observed NPs were spaced far from each other, and none of them accumulated even after 12 h of incubation. These results suggest that the NPs were free from protein aggregations and did not reside on the plasma membranes around the nuclei.

## Discussion

This study used an approach combining *d*STORM, transcriptomics, and molecular biology to assess the genotoxicity of NPs in in vitro. These findings provide important insights for predicting the potential nanotoxicity at the cellular level.

Intracellular aggregation presents the major concern that arises when detecting MNPs*@*SiO_2_(RITC) in single cells with imaging-fluorescence-labeled NPs. Given diffraction limitations, the task of distinguishing individual NPs in the aggregated regions of cells proves to be difficult. Therefore, the labeled dye should be of sufficient quality to support super-resolution imaging for distinguishing individual MNPs*@*SiO_2_(RITC) for performing the accurate site-specific quantitative estimation of the internalized NPs [52]. The resulting super-resolution images obtained with *d*STORM enabled individual MNPs*@*SiO_2_(RITC) in the aggregated region to be distinguished and provided improved resolution over the results obtained from conventional wide-field TIRF images.

On incubation, the NPs were widely sensed and internalized by macrophage cells. The macrophage cells are well known for their phagocytic uptake of particles [53]. They can efficiently uptake particles by expressing several receptors on the cell surface to recognize foreign particles and molecules [54, 55]. However, the optimum size for phagocytosis was comparatively large, around 250 nm to 3 μm [11]. Endocytosis, unlike phagocytosis, occurred in all types of cells and was restricted to about 120 nm. Larger diameter particles could not be taken up by endocytosis [56]. As we used the 50 nm MNPs*@*SiO_2_(RITC), the uptake of these NPs largely depended on endocytosis rather than phagocytosis. The buffer medium was the other factor that could influence NP uptake [53, 56]. There was no significant difference in the number of NPs internalized when using media or PBS.

Earlier studies of MNPs*@*SiO_2_(RITC) internalization did not find NPs internalized in the nuclear region of cells when *z*-sectional images confocal microscopy [3, 45, 57] was used. The discrepancies in the present results can be attributed to the advantages of super-resolution imaging over site-specific analysis. The MNPs*@*SiO_2_(RITC) internalization in nuclei was attributed to the nuclear pore size of the cells [58, 59]. However, exceptions appeared where NPs with sizes greater than 70 nm were reported to be internalized in the nuclear regions of cells [60]. Further studies are needed to elucidate the mechanism of internalization in the nuclear regions of cells.

Herein, we observed 50 nm MNPs*@*SiO_2_(RITC) internalized in the cell nucleus region in both RAW 264.7 and HEK293 cells. The kinetics of internalization varied by cell type. RAW 264.7 cells consumed more than both HEK293 and NIH3T3 cells. The NPs were internalized in RAW 264.7 cells only after 5 h of incubation; meanwhile, 12 h of incubation were required by HEK293 cells. NIH3T3 cells showed high resistance towards NPs. After 12 h of incubation, there was still no sign of a fluorescence signal in the cell nuclei of NIH3T3 cells. DNA repair-related gene disturbances were analyzed in HEK293 cells and micronuclei were detected in both HEK293 and RAW 264.7 cells, indicating genotoxicity. In addition, cell viability was significantly decreased in MNPs@SiO_2_(RITC)-treated RAW 264.7 cells in a dose-dependent manner. Moreover, MNPs*@*SiO_2_(RITC) showed high levels of uptake in RAW 264.7 cells, as compared to HEK293 and NIH3T3 cells. Because the increase in reactive oxygen species (ROS) generation by MNPs@SiO_2_(RITC) in cells is linked to cell viability [45], these results are consistent with data of cell viability previous reported: RAW 264.7 cells, 20–40%; A549 cells, ~ 70% [50].

We also evaluated the cellular uptake of MNPs@SiO_2_(RITC) and SiO_2_ NPs in three types of primary cells (Kupffer cells, hepatocytes, and liver sinusoidal endothelial cells) from the liver, one of the major organs for NP accumulation [61–63]. The highest MNPs@SiO_2_(RITC) internalization was observed in hepatocytes, followed by Kupffer cells and liver sinusoidal endothelial cells, as indicated by the intensity of MNPs@SiO_2_(RITC) (Additional file 1: Figs. S15, S16). SiO_2_ NPs internalizations also showed a similar trend (hepatocytes > Kupffer cells > liver sinusoidal endothelial cells) of MNPs@SiO_2_(RITC) in 10, 50, and 200-nm SiO_2_ NPs treated cells (Additional file 1: Figs. S17–S22). Furthermore, the highest SiO_2_ NPs internalization was observed in 10-nm SiO_2_ NPs treated cells, followed by 50 and 200-nm SiO_2_ NPs, as indicated by the intensity of the SiO_2_ NPs. Moreover, cell viability decreased in a SiO_2_ NPs internalization level-dependent manner (Additional file 1: Fig. S23). However, there was a discrepancy between these results and those of the in vivo experiments; the highest NP internalization was observed in Kupffer cells, followed by liver sinusoidal endothelial cells, and hepatocytes [64]. Thus, we suppose that this discrepancy between in vitro and in vivo conditions may be the result of a difference in the surface area among the cells and organ structure-dependent exposure availability.

Changes in DNA repair-related transcriptome and formation of micronuclei, which are responses and phenomena related to DNA damage [65, 66], were observed in MNPs@SiO_2_(RITC) treated cells. Although the number of NPs that entered the cell nuclei of RAW 264.7 and HEK293 cells was much lower than the total number of NPs in the cells, that number was sufficient for generating genotoxicity in the cells. Thus, the presence of NPs in the cell nuclei may induce dysfunctions in the nuclei and genotoxicity through the aggregation of intranuclear proteins or inhibition of RNA transcription. In addition, previous studies suggested that these biological effects of MNPs@SiO_2_(RITC) were caused by the silica shell rather than the cobalt ferrite core [42, 43, 45–47, 67], because there was no release of free iron in the intracellular environment from SiO_2_-coated Fe_3_O_4_ NPs induced ROS after 48 h in the cells [45].

## Conclusions

The major drawbacks of using engineered NPs as drug and gene carriers are the unknown influences on in vivo toxicity, pharmacokinetics, and intracellular NP internalization. In this study, cell viability was evaluated by intracellular MNPs@SiO_2_(RITC) using the MTS assay, and a single particle tracking approach was performed by visualizing the interaction of MNPs@SiO_2_(RITC) particles with the cell membrane using *d*STORM. Subdiffraction-limit spatial resolution resolved individual MNPs@SiO_2_(RITC) and resulted in the quantification of MNPs@SiO_2_(RITC) in specific regions of the cells. Quantitative analysis suggests that macrophage cells are more susceptible to MNPs@SiO_2_(RITC) than human kidney cells and fibroblast cells at the point of NP uptake efficiency. Moreover, the MNPs@SiO_2_(RITC) were observed to be internalized in the nuclear regions of the cells by breaking the biological barriers. Thus, the movement was monitored, and individual NPs were tracked with ultra-high spatial resolution at the subdiffraction limit. Further studies, evaluating the mechanism of subcellular localization of MNPs@SiO_2_(RITC) in NP-treated cells, will help improve predictions of nanotoxicity and aid in the development of NPs as diagnostic and therapeutic agents.

## Supplementary Information

**Additional file 1: Table S1.** Ingenuity Pathway Analysis-based profiles of DNA repair-related genes in HEK293 cells treated with MNPs@SiO_2_(RITC). **Table S2**. ICP-QMS and AAS data for Co and Fe quantification in 3 × 10^6^ cells of HEK293, NIH3T3 and RAW 264.7 at 0.01 µg/mL MNPs@SiO_2_(RITC). **Table S3**. Quantitative real time PCR primer sequences for genes encoding DNA repair related genes. **Fig. S1**. Characterization of mouse primary liver cells. a Morphological analysis of isolated Kupffer cells, hepatocytes, and liver sinusoidal endothelial cells. Scale bar = 20 μm. Cell specific marker protein expression analysis for b F4/80 using flow cytometry, c albumin, and CD31 using immunoblotting. β-actin was used as the internal control. **Fig. S2.** (a) Physical layout and (b) schematic representation of the lab-made *d*STORM setup. Following acronyms were used; L, laser; M, mirror; DCM, dichroic mirror; MS, mechanical shutter; NP, Nomarski prism; EM-CCD, electron-multiplying cooled charge-coupled device. **Fig. S3.** Plot of drift correction on the *x*- and *y*-axes with respect to frame illustrated with a fiducial marker. Images of raw, before drift correction and after drift correction. **Fig. S4.** a. The photo-switching rate constant *k*_off_ was obtained by irradiation of 532 nm laser and monitoring the decrease in the fluorescence intensity over time. b. The photo-switching rate constant *k*_on_ was obtained by simultaneous irradiation of the sample with 405 nm and 532 nm (200 mW/cm^2^) and monitoring the increase in the fluorescence intensity with time. Both *k*_off_ and *k*_on_ shows a linear dependence with illuminated intensities. Error bars represent the standard deviations of five measurements. c. Photoinduced restoration of RITC to fluorescence state with 405 nm activation laser. **Fig. S5.** Wide-field and *d*STORM images of HEK293, NIH3T3, and RAW 264.7 cells incubated for 1 h with three different concentrations a. 0.1 µg/mL, b. 0.01 µg/mL, and c. 0.003 µg/mL of 50-nm MNPs*@*SiO_2_(RITC). **Fig. S6.** Wide-field and *d*STORM images of HEK293, NIH3T3, and RAW 264.7 cells incubated for 3 h with three different concentrations a. 0.1 µg/mL, b. 0.01 µg/mL, and c. 0.003 µg/mL of 50-nm MNPs*@*SiO_2_(RITC). **Fig. S7.** The wide-field TIRF images of Fig. 1b. A single emitter is magnified and the consecutive images show the photo-switching events in the same spot and the corresponding Gaussian fitting for localizing the centroid of the individual NPs within diffraction-limited images. **Fig. S8.** The wide-field TIRF images of the RAW 264.7 cell incubated with 0.01 µg/mL of 50-nm MNPs*@*SiO_2_(RITC) for 5 h with a. serum free DMEM and b. PBS buffer. **Fig. S9.** Evaluation of cytotoxicity in MNPs@SiO_2_(RITC)-treated cells. MTS assay of MNPs@SiO_2_(RITC)-treated HEK293, NIH3T3, and RAW 264.7 cells. Cells were treated with 0.1 µg/mL, 0.3 µg/mL and 1.0 µg/mL MNPs@SiO_2_(RITC) for 12 h, followed by cell viability assessment using MTS assay. **Fig. S10.** Sixty-nine DNA-repair-related genes were constructed algorithmically using Ingenuity Pathway Analysis (IPA). Red and green in the genetic network indicate up- and down-regulated genes, respectively, in HEK293 cells treated with 1.0 mg/mL MNPs@SiO_2_(RITC), when compared with non-treated controls for 12 h. The data set of differentially expressed genes obtained from microarray data with a greater than threefold change is shown. **Fig. S11.** Sixty-nine DNA repair related genes showing significantly altered expression by MNPs@SiO_2_(RITC) on microarray analysis. DNA repair-related genes were constructed algorithmically using IPA. Red and green in the genetic network indicate up- and down-regulated genes, respectively, in HEK293 cells treated with 0.1 mg/mL MNPs@SiO_2_(RITC), when compared with non-treated controls for 12 h. The data set of differentially expressed genes obtained from microarray data with > threefold change is shown. Thirty-six genes were significantly changed. **Fig. S12.** DIC, wide-field, and *d*STORM images of HEK293, NIH3T3, and RAW 264.7 cell respectively. Images were acquired after incubation of 50-nm MNPs*@*SiO_2_(RITC) for 3 h with each cell. Merged images of DIC and *d*STORM for site-specific analysis of the internalized NPs. **Fig. S13.** DIC, wide-field, and *d*STORM images of HEK293, NIH3T3, and RAW 264.7 cell respectively. Images were acquired after incubation of 50-nm MNPs*@*SiO_2_(RITC) for 5 h with each cell. Merged images of DIC and *d*STORM for site-specific analysis of the internalized NPs. **Fig. S14.** DIC, wide-field, and *d*STORM images of HEK293, NIH3T3, and RAW 264.7 cell respectively. Images were acquired after incubation of 50-nm MNPs*@*SiO_2_(RITC) for 12 h with each cell. Merged images of DIC and *d*STORM for site-specific analysis of the internalized NPs. **Fig. S15.** DIC and super-resolution images of Kupffer cells, hepatocytes, and liver sinusoidal endothelial cells incubated for 5 h with 2 µg/mL concentration of 50-nm MNPs@SiO_2_(RITC). **Fig. S16.** DIC and super-resolution images of Kupffer cells, hepatocytes, and liver sinusoidal endothelial cells incubated for 24 h with 2 µg/mL concentration of 50-nm MNPs*@*SiO_2_(RITC). **Fig. S17.** DIC and super-resolution images of Kupffer cells, hepatocytes, and liver sinusoidal endothelial cells incubated for 5 h with 2 µg/mL concentration of 10-nm SiO_2_ NPs. **Fig. S18.** DIC and super-resolution images of Kupffer cells, hepatocytes, and liver sinusoidal endothelial cells incubated for 5 h with 2 µg/mL concentration of 50-nm SiO_2_ NPs. **Fig. S19.** DIC and super-resolution images of Kupffer cells, hepatocytes, and liver sinusoidal endothelial cells incubated for 5 h with 2 µg/mL concentration of 200-nm SiO_2_ NPs. **Fig. S20.** DIC and super-resolution images of Kupffer cells, hepatocytes, and liver sinusoidal endothelial cells incubated for 24 h with 2 µg/mL concentration of 10-nm SiO_2_ NPs. **Fig. S21.** DIC and super-resolution images of Kupffer cells, hepatocytes, and liver sinusoidal endothelial cells incubated for 24 h with 2 µg/mL concentration of 50-nm SiO_2_ NPs. **Fig. S22.** DIC and super-resolution images of Kupffer cells, hepatocytes, and liver sinusoidal endothelial cells incubated for 24 h with 2 µg/mL concentration of 200-nm SiO_2_ NPs. **Fig. S23.** Evaluation of cytotoxicity in SiO_2_ NPs-treated cells. MTS assay of a. 10-nm, b. 50-nm, and c. 200-nm SiO_2_ NPs-treated Kupffer cells, hepatocytes, and liver sinusoidal endothelial cells. Cells were treated with 0.1 mg/mL, 0.3 mg/mL and 1.0 mg/mL SiO_2_ NPs for 12 h, followed by cell viability assessment using MTS assay.

**Additional file 2: Movie S1.** Photo-switching of 50-nm MNPs*@*SiO_2_(RITC) internalized in the HEK293 cell after 5 h of incubation.

**Additional file 3: Movie S2.** 2D and 3D wide-field fluorescence and DIC imaging of 50-nm MNPs@SiO_2_(RITC) internalized in RAW 264.7 cell.

**Additional file 4: Movie S3.** wide-field fluorescence (left) and DIC merged wide-field fluorescence image (right) of real-time dynamics corresponds to 2000 frames of 25000 frames with 50-nm MNPs*@*SiO_2_(RITC) internalized in the cell nucleus of RAW 264.7 cell.

## Data Availability

The data supporting the findings of this study are available from the corresponding author, upon reasonable request.

## Abbreviations

2D: Two-dimensional
AAS: Atomic absorption spectrometer
ANOVA: One-way analysis of variance
*CD31*: Cluster of differentiation 31
*CRACR2A*: Calcium release activated channel regulator 2A
*DAZAP2*: DAZ associated protein 2
DIC: Differential interference contrast
*d*STORM: Direct stochastic optical reconstruction microscopy
EM-CCD: Electron-multiplying cooled charge-coupled device camera
F4/80: Protein encoded in adhesion G protein coupled receptor E1 (*ADGRE1*) gene
FBS: Fetal bovine serum
*HERPUD1*: Homocysteine-inducible, endoplasmic reticulum stress-inducible, ubiquitin-like domain member 1
ICP-QMS: Inductively coupled plasma/quadrupole mass spectrometer
IPA: Ingenuity pathway analysis
MEA: *β*-Mercaptoethylamine
MNPs: Magnetic nanoparticles
MRI: Magnetic resonance imaging
MTS: (3-(4,5-Dimethylthiazol-2-yl)-5-(3-carboxymethoxyphenyl)-2-(4-sulfophenyl)-2H-tetrazolium)
N.I.: Normalized intensity
NP: Nanoparticle
PBS: Phosphate-buffered saline
PALM: Photoactivated localization microscopy
*PECAM-1*: Platelet endothelial cell adhesion molecule
PCR: Polymerase chain reaction
PLL: Poly-l-lysine
*REST*: RE1-silencing transcription factor
RITC: Rhodamine B isothiocyanate
ROS: Reactive oxygen species
SIM: Structured-illumination microscopy
STED: Stimulated-emission depletion
TIRF: Total internal reflection fluorescence
*UBQLN4*: Ubiquilin 4
XRD: X-ray diffraction
