# Supplementary material for: Quantifying intracellular trafficking of silica-coated magnetic nanoparticles in live single cells by site-specific direct stochastic optical reconstruction microscopy
Source: J Nanobiotechnology. 2021 Nov 29;19:398. doi: 10.1186/s12951-021-01147-1 (PMC8628397; doi:10.1186/s12951-021-01147-1)
Supplement: Supplementary file 1 — Additional file 1: Table S1. Ingenuity Pathway Analysis-based profiles of DNA repair-related genes in HEK293 cells treated with MNPs@SiO2(RITC). Table S2. ICP-QMS and AAS data for Co and Fe quantification in 3 × 10^6^ cells of HEK293, NIH3T3 and RAW 264.7 at 0.01 µg/mL MNPs@SiO2(RITC). Table S3. Quantitative real time PCR primer sequences for genes encoding DNA repair related genes. Fig. S1. Characterization of mouse primary liver cells. a Morphological analysis of isolated Kupffer cells, hepatocytes, and liver sinusoidal endothelial cells. Scale bar = 20 μm. Cell specific marker protein expression analysis for b F4/80 using flow cytometry, c albumin, and CD31 using immunoblotting. β-actin was used as the internal control. Fig. S2. (a) Physical layout and (b) schematic representation of the lab-made dSTORM setup. Following acronyms were used; L, laser; M, mirror; DCM, dichroic mirror; MS, mechanical shutter; NP, Nomarski prism; EM-CCD, electron-multiplying cooled charge-coupled device. Fig. S3. Plot of drift correction on the x- and y-axes with respect to frame illustrated with a fiducial marker. Images of raw, before drift correction and after drift correction. Fig. S4. a. The photo-switching rate constant koff was obtained by irradiation of 532 nm laser and monitoring the decrease in the fluorescence intensity over time. b. The photo-switching rate constant kon was obtained by simultaneous irradiation of the sample with 405 nm and 532 nm (200 mW/cm2) and monitoring the increase in the fluorescence intensity with time. Both koff and kon shows a linear dependence with illuminated intensities. Error bars represent the standard deviations of five measurements. c. Photoinduced restoration of RITC to fluorescence state with 405 nm activation laser. Fig. S5. Wide-field and dSTORM images of HEK293, NIH3T3, and RAW 264.7 cells incubated for 1 h with three different concentrations a. 0.1 µg/mL, b. 0.01 µg/mL, and c. 0.003 µg/mL of 50-nm MNPs@SiO2(RITC). Fig. S6. Wide-field an [file 12951_2021_1147_MOESM1_ESM.docx]

**Additional File 1**

**Quantifying intracellular trafficking of silica-coated magnetic nanoparticles in live single cells by site-specific direct stochastic optical reconstruction microscopy**

**Suresh Kumar Chakkarapani**^#^ (sureshsrkmv@gmail.com)

Department of Chemistry, Graduate School, Kyung Hee University, Yongin-si, Gyeonggi-do 17104, Republic of Korea

**Tae Hwan Shin**^#^ (catholicon@ajou.ac.kr)

Department of Physiology, Ajou University School of Medicine, Suwon-si, Gyeonggi-do 16499, Republic of Korea

**Seungah Lee**^#^ (moon11311@naver.com)

Department of Applied Chemistry and Institute of Natural Sciences, Kyung Hee University, Yongin-si, Gyeonggi-do 17104, Republic of Korea

**Kyung-Soo Park** (pks6475@kist.re.kr)

Nanophotonics Research Center, Korea Institute of Science and Technology, Seoul 02792, Republic of Korea

**Gwang Lee*** (glee@ajou.ac.kr)

Department of Molecular Science and Technology, Ajou University, Suwon-si, Gyeonggi-do 16499, Republic of Korea and Department of Physiology, Ajou University School of Medicine, Suwon-si, Gyeonggi-do 16499, Republic of Korea

**Seong Ho Kang*** (shkang@khu.ac.kr)

Department of Chemistry, Graduate School, Kyung Hee University, Yongin-si, Gyeonggi-do 17104, Republic of Korea and Department of Applied Chemistry and Institute of Natural Sciences, Kyung Hee University, Yongin-si, Gyeonggi-do 17104, Republic of Korea

^#^These authors contributed equally to this work.

*Corresponding authors:

Gwang Lee, Professor

Department of Physiology, Ajou University School of Medicine, 164, World cup-ro, Yeongtong-gu, Suwon-si, Gyeonggi-do 16499, Republic of Korea

Phone: +82-31-219-4554

Fax: +82-31-219-5049

E-mail: glee@ajou.ac.kr

Seong Ho Kang, Professor

Department of Applied Chemistry and Institute of Natural Sciences, Kyung Hee University, Yongin-si, Gyeonggi-do 17104, Republic of Korea

Phone: +82-31-201-3349

Fax: +82-31-201-2340

E-mail: shkang@khu.ac.kr

**Table S1.** Quantitative real time PCR primer sequences for genes encoding DNA repair related genes.

| **Gene name** | **Symbol** | **NCBI Ref. seq** | **Direction** | **Primer sequence**  **(5′-3′)** |
| --- | --- | --- | --- | --- |
| Homo sapiens calcium release activated channel regulator 2A | *CRACR2A* | NM_001144958.1 | Forward | AGC TGA AAC TCA CTA ACC AGG |
|  |  |  | Reverse | CAC ACG GTA CAC TTC CAT CTC |
| Homo sapiens RE1-silencing transcription factor | *REST* | BC132859.1 | Forward | GGC CAG TTC CAC AAG AAT CTA G |
|  |  |  | Reverse | ACA AAA TCT CCC TTA GCC GC |
| Homo sapiens DAZ associated protein 2 | *DAZAP2* | BC002334.2 | Forward | CAT CCC ATC TCC AAC CCT AG |
|  |  |  | Reverse | CTT TCT GTC CTG CAC CAT TG |
| Homo sapiens homocysteine-inducible, endoplasmic reticulum stress-inducible, ubiquitin-like domain member 1 | *HERPUD1* | BC000086.1 | Forward | TGG AGG CTT TGA CAG GAA TG |
|  |  |  | Reverse | GGC TTC ACG TTT CTG CTT TTG |
| Homo sapiens ubiquilin 4 | *UBQLN4* | BC063841.1 | Forward | CTG TCA ATC CTT ACC CTC TGC |
|  |  |  | Reverse | CAT GCC TCT AAA GTC ACC CTG |
| Homo sapiens glyceraldehyde 3-phosphate dehydrogenase | *GAPDH* | NM_002046 | Forward | GAA GAC TGT GGA TGG CCC |
|  |  |  | Reverse | CCA TGC CAG TGA GCT TCC |

Ref. seq.: Reference sequence.

**Table S2**. Ingenuity Pathway Analysis-based profiles of DNA repair-related genes in HEK293 cells treated with MNPs@SiO_2_(RITC).

| Entrez gene name | Symbol | Affymetrix ID | Location | Signal fold change^a^ | |
| --- | --- | --- | --- | --- | --- |
|  |  |  |  | 0.1 mg/mL | 1.0 mg/mL |
| adipogenesis regulatory factor | *ADIRF* | 203571_s_at | Nucleus | -3.97 | -3.96 |
| adrenoceptor alpha 1B | *ADRA1B* | 207589_at | Plasma Membrane | -6.81 | -7.72 |
| chromosome 14 open reading frame 1 | *C14orf1* | 202563_at | Cytoplasm | -9.70 | -4.37 |
| coiled-coil domain containing 7 | *CCDC7* | 1553214_a_at | Other | -12.54 | -22.78 |
| CDK5 regulatory subunit associated protein 2 | *CDK5RAP2* | 243153_at | Cytoplasm | 1.50 | 5.89 |
| cofilin 1 (non-muscle) | *CFL1* | 236792_at | Nucleus | 5.92 | 3.24 |
| ceroid-lipofuscinosis, neuronal 8 | *CLN8* | 229958_at | Cytoplasm | 4.51 | -24.41 |
| COX17, cytochrome c oxidase copper chaperone | *COX17* | 1558346_at | Cytoplasm | 5.15 | 5.74 |
| calcium release activated channel regulator 2A | *CRACR2A* | 228752_at | Cytoplasm | -2.76 | -3.52 |
| carnitine O-octanoyltransferase | *CROT* | 243283_at | Cytoplasm | 13.29 | 3.37 |
| cytochrome b561 family member D2 | *CYB561D2* | 229636_at | Other | -4.91 | -4.95 |
| cytochrome b5 type B (outer mitochondrial membrane) | *CYB5B* | 238554_at | Cytoplasm | 1.91 | -3.88 |
| DAZ associated protein 2 | *DAZAP2* | 238300_s_at | Nucleus | 2.29 | 3.92 |
| DEAH-box helicase 34 | *DHX34* | 204816_s_at | Other | 1.73 | -3.63 |
| EWS RNA binding protein 1 | *EWSR1* | 210012_s_at | Nucleus | 12.38 | 4.23 |
| exonuclease 3'-5' domain containing 3 | *EXD3* | 1555914_a_at | Other | 1.65 | 5.83 |
| fatty acid 2-hydroxylase | *FA2H* | 234963_s_at | Cytoplasm | 1.50 | -5.74 |
| free fatty acid receptor 1 | *FFAR1* | 231761_at | Plasma Membrane | -3.61 | -7.66 |
| formin like 3 | *FMNL3* | 238823_at | Cytoplasm | 7.25 | -24.37 |
| growth associated protein 43 | *GAP43* | 216967_at | Plasma Membrane | -14.34 | 3.71 |
| glycine-N-acyltransferase | *GLYAT* | 222083_at | Cytoplasm | -3.94 | -4.25 |
| golgin A7 family member B | *GOLGA7B* | 228068_at | Other | -11.99 | -9.78 |
| HMG-box transcription factor 1 | *HBP1* | 236645_at | Nucleus | 17.10 | 7.01 |
| homocysteine-inducible, endoplasmic reticulum stress-inducible, ubiquitin-like domain member 1 | *HERPUD1* | 1569380_a_at | Cytoplasm | 1.28 | 3.74 |
| histone cluster 1, H3e | *HIST1H3E* | 214616_at | Nucleus | 4.24 | 12.99 |
| histone cluster 1, H4f | *HIST1H4F* | 234960_at | Nucleus | 2.55 | -6.14 |
| HNF1 homeobox A | *HNF1A* | 216930_at | Nucleus | 1.60 | -4.21 |
| potassium channel, voltage gated eag related subfamily H, member 6 | *KCNH6* | 221023_s_at | Plasma Membrane | 4.06 | 5.71 |
| potassium channel regulator | *KCNRG* | 239098_at | Cytoplasm | 3.98 | 3.33 |
| KRR1, small subunit (SSU) processome component, homolog (yeast) | *KRR1* | 235038_at | Nucleus | 22.26 | -14.59 |
| mitogen-activated protein kinase kinase kinase kinase 2 | *MAP4K2* | 204936_at | Cytoplasm | 2.74 | 5.76 |
| mitogen-activated protein kinase 8 interacting protein 2 | *MAPK8IP2* | 1556001_at | Cytoplasm | -2.64 | 4.39 |
| mitochondrial ribosomal protein S12 | *MRPS12* | 213840_s_at | Cytoplasm | -1.46 | -6.01 |
| neurofilament, heavy polypeptide | *NEFH* | 33767_at | Cytoplasm | -1.59 | -10.10 |
| Na+/K+ transporting ATPase interacting 2 | *NKAIN2* | 242002_at | Plasma Membrane | -5.24 | -4.02 |
| ORAI calcium release-activated calcium modulator 2 | *ORAI2* | 230347_at | Plasma Membrane | -1.70 | -11.42 |
| pancreatic and duodenal homeobox 1 | *PDX1* | 208559_at | Nucleus | -12.58 | -3.78 |
| protein phosphatase 2 regulatory subunit B, gamma | *PPP2R2C* | 228140_s_at | Other | 5.34 | 10.26 |
| RAB1A, member RAS oncogene family | *RAB1A* | 239570_at | Cytoplasm | 1.35 | 3.11 |
| RAB37, member RAS oncogene family | *RAB37* | 228113_at | Cytoplasm | 6.85 | 4.42 |
| RNA binding motif protein 48 | *RBM48* | 221594_at | Nucleus | -6.52 | -11.52 |
| RE1-silencing transcription factor | *REST* | 204536_s_at | Nucleus | -3.99 | -7.54 |
| raftlin, lipid raft linker 1 | *RFTN1* | 230260_s_at | Plasma Membrane | 4.38 | 7.04 |
| rhabdomyosarcoma 2 associated transcript (non-protein coding) | *RMST* | 229782_at | Other | -3.84 | -4.06 |
| roundabout guidance receptor 2 | *ROBO2* | 226766_at | Plasma Membrane | -1.73 | -4.90 |
| ribosomal protein S6 kinase, 90kDa, polypeptide 5 | *RPS6KA5* | 1554319_at | Nucleus | 7.47 | 4.27 |
| ribosomal protein S6 kinase, 90kDa, polypeptide 6 | *RPS6KA6* | 220738_s_at | Cytoplasm | 6.97 | 5.23 |
| ribosomal RNA processing 15 homolog | *RRP15* | 219037_at | Nucleus | 2.05 | -3.01 |
| squamous cell carcinoma antigen recognized by T-cells 3 | *SART3* | 1554276_at | Nucleus | -1.21 | 3.40 |
| SEC24 homolog A, COPII coat complex component | *SEC24A* | 212900_at | Cytoplasm | 1.50 | -5.19 |
| serpin peptidase inhibitor, clade I (pancpin), member 2 | *SERPINI2* | 207636_at | Extracellular Space | 2.42 | -3.57 |
| seizure related 6 homolog (mouse)-like | *SEZ6L* | 231650_s_at | Plasma Membrane | -18.66 | -8.20 |
| solute carrier family 13 (sodium/sulfate symporter), member 1 | *SLC13A1* | 220502_s_at | Plasma Membrane | 3.49 | 23.71 |
| solute carrier family 38 member 4 | *SLC38A4* | 220786_s_at | Plasma Membrane | 14.96 | 20.34 |
| solute carrier family 7 member 14 | *SLC7A14* | 232904_at | Cytoplasm | -1.36 | 12.65 |
| StAR related lipid transfer domain containing 9 | *STARD9* | 227108_at | Cytoplasm | -1.39 | -4.37 |
| Sad1 and UNC84 domain containing 2 | *SUN2* | 229548_at | Nucleus | 2.49 | 3.97 |
| spectrin repeat containing, nuclear envelope 1 | *SYNE1* | 232027_at | Nucleus | 9.20 | 11.01 |
| spectrin repeat containing, nuclear envelope 2 | *SYNE2* | 202761_s_at | Nucleus | 1.49 | 3.38 |
| TRK-fused gene | *TFG* | 239385_at | Cytoplasm | 1.51 | 3.52 |
| transmembrane protein 107 | *TMEM107* | 235490_at | Other | -1.80 | -3.02 |
| ubiquilin 4 | *UBQLN4* | 222252_x_at | Cytoplasm | 1.26 | 3.43 |
| unc-119 lipid binding chaperone | *UNC119* | 203271_s_at | Cytoplasm | 2.30 | 3.34 |
| uridine phosphorylase 2 | *UPP2* | 233155_at | Cytoplasm | -2.53 | -3.28 |
| ubiquitin specific peptidase 25 | *USP25* | 1563497_at | Cytoplasm | 3.24 | 3.05 |
| ubiquitin specific peptidase 44 | *USP44* | 224048_at | Nucleus | 1.68 | -3.87 |
| X-linked Kx blood group related 4 | *XKR4* | 230692_at | Other | 21.38 | 34.59 |
| ZFP64 zinc finger protein | *ZFP64* | 229186_s_at | Nucleus | 1.37 | -6.17 |
| zinc finger protein 775 | *ZNF775* | 230517_at | Other | -6.75 | -9.20 |

^a^Normalized signal fold change of signal in treated groups with MNPs@SiO_2_(RITC) to corresponding signal of in control group.

**Table S3**. ICP-QMS and AAS data for Co and Fe quantification in 3 × 10^6^ cells of HEK293, NIH3T3 and RAW 264.7 at 0.01 µg/mL MNPs@SiO_2_(RITC).

| Cell type | Region | Co (ppb)^a^ | Fe (ppm)^b^ |
| --- | --- | --- | --- |
| HEK 293 | Whole cell | <12.5 | <5 |
|  | Nucleus | <12.5 | <5 |
| NIH3T3 | Whole cell | <12.5 | <5 |
|  | Nucleus | 41.8 | <5 |
| RAW 264.7 | Whole cell | <12.5 | <5 |
|  | Nucleus | 63.8 | <5 |

^a^12.5 signifies limit of detection of ICP/QMS

^b^5 is the limit of detection of AAS.


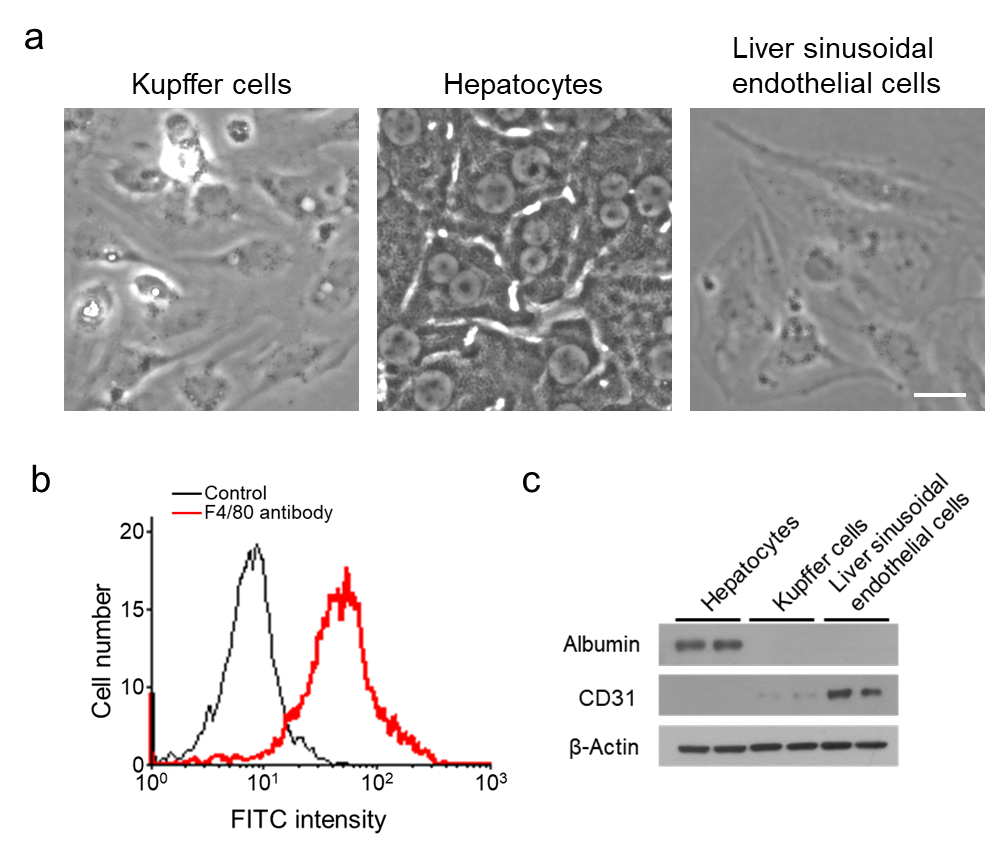


**Fig. S1.** Characterization of mouse primary liver cells. **a** Morphological analysis of isolated Kupffer cells, hepatocytes, and liver sinusoidal endothelial cells. Scale bar = 20 μm. Cell specific marker protein expression analysis for **b** F4/80 using flow cytometry, **c** albumin, and CD31 using immunoblotting. β-actin was used as the internal control.

**Fig. S2.** **a** Physical layout and **b** schematic representation of the lab-made *d*STORM setup. Following acronyms were used; L, laser; M, mirror; DCM, dichroic mirror; MS, mechanical shutter; NP, Nomarski prism; EM-CCD, electron-multiplying cooled charge-coupled device.

**Fig. S3.** Plot of drift correction on the *x*- and *y*-axes with respect to frame illustrated with a fiducial marker. Images of raw, before drift correction and after drift correction.

**Fig. S4.** **a** The photo-switching rate constant *k*_off_ was obtained by irradiation of 532 nm laser and monitoring the decrease in the fluorescence intensity over time. **b** The photo-switching rate constant *k*_on_ was obtained by simultaneous irradiation of the sample with 405 nm and 532 nm (200 mW/cm^2^) and monitoring the increase in the fluorescence intensity with time. Both *k*_off_ and *k*_on_ shows a linear dependence with illuminated intensities. Error bars represent the standard deviations of five measurements. **c** Photoinduced restoration of RITC to fluorescence state with 405 nm activation laser.

**Fig. S5.** Wide-field and *d*STORM images of HEK293, NIH3T3, and RAW 264.7 cells incubated for 1 h with three different concentrations **a** 0.1 µg/mL, **b** 0.01 µg/mL, and **c** 0.003 µg/mL of 50-nm MNPs*@*SiO_2_(RITC).

**Fig. S6.** Wide-field and *d*STORM images of HEK293, NIH3T3, and RAW 264.7 cells incubated for 3 h with three different concentrations **a** 0.1 µg/mL, **b** 0.01 µg/mL, and **c** 0.003 µg/mL of 50-nm MNPs*@*SiO_2_(RITC).

**Fig. S7.** The wide-field TIRF images of Fig. 1b. A single emitter is magnified and the consecutive images show the photo-switching events in the same spot and the corresponding Gaussian fitting for localizing the centroid of the individual NPs within diffraction-limited images.

**Fig. S8.** The wide-field TIRF images of the RAW 264.7 cell incubated with 0.01 µg/mL of 50-nm MNPs*@*SiO_2_(RITC) for 5 h with **a** serum free DMEM and **b** PBS buffer.

**Fig. S9.** Evaluation of cytotoxicity in MNPs@SiO_2_(RITC)-treated cells. MTS assay of MNPs@SiO_2_(RITC)-treated HEK293, NIH3T3, and RAW 264.7 cells. Cells were treated with 0.1 µg/mL, 0.3 µg/mL and 1.0 µg/mL MNPs@SiO_2_(RITC) for 12 h, followed by cell viability assessment using MTS assay.

**Fig. S10.** Sixty-nine DNA-repair-related genes were constructed algorithmically using Ingenuity Pathway Analysis (IPA). Red and green in the genetic network indicate up- and down-regulated genes, respectively, in HEK293 cells treated with 1.0 mg/mL MNPs@SiO_2_(RITC), when compared with non-treated controls for 12 h. The data set of differentially expressed genes obtained from microarray data with a greater than threefold change is shown.

**Fig. S11.** Sixty-nine DNA repair related genes showing significantly altered expression by MNPs@SiO_2_(RITC) on microarray analysis. DNA repair-related genes were constructed algorithmically using Ingenuity Pathway Analysis (IPA). Red and green in the genetic network indicate up- and down-regulated genes, respectively, in HEK293 cells treated with 0.1 mg/mL MNPs@SiO_2_(RITC), when compared with non-treated controls for 12 h. The data set of differentially expressed genes obtained from microarray data with > 3-fold change is shown. Thirty-six genes were significantly changed.

**Fig. S12.** DIC, wide-field, and *d*STORM images of HEK293, NIH3T3, and RAW 264.7 cell respectively. Images were acquired after incubation of 50-nm MNPs*@*SiO_2_(RITC) for 3 h with each cell. Merged images of DIC and *d*STORM for site-specific analysis of the internalized NPs.

**Fig. S13.** DIC, wide-field, and *d*STORM images of HEK293, NIH3T3, and RAW 264.7 cell respectively. Images were acquired after incubation of 50-nm MNPs*@*SiO_2_(RITC) for 5 h with each cell. Merged images of DIC and *d*STORM for site-specific analysis of the internalized NPs.

**Fig. S14.** DIC, wide-field, and *d*STORM images of HEK293, NIH3T3, and RAW 264.7 cell respectively. Images were acquired after incubation of 50-nm MNPs*@*SiO_2_(RITC) for 12 h with each cell. Merged images of DIC and *d*STORM for site-specific analysis of the internalized NPs.

**Fig. S15.** DIC and super-resolution images of Kupffer cells, hepatocytes, and liver sinusoidal endothelial cells incubated for 5 h with 2 µg/mL concentration of 50-nm MNPs@SiO_2_(RITC).

**Fig. S16.** DIC and super-resolution images of Kupffer cells, hepatocytes, and liver sinusoidal endothelial cells incubated for 24 h with 2 µg/mL concentration of 50-nm MNPs*@*SiO_2_(RITC).


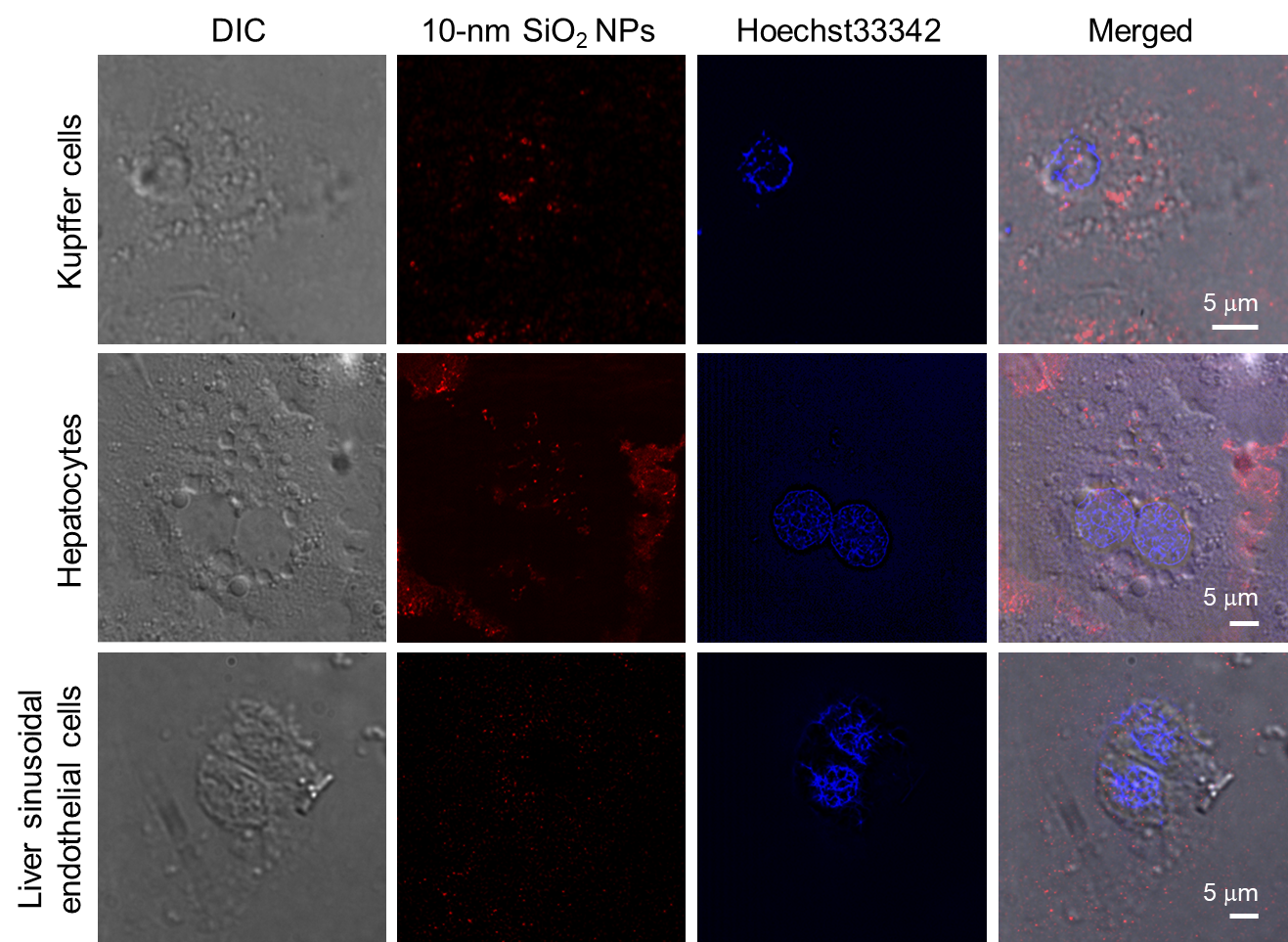


**Fig. S17.** DIC and super-resolution images of Kupffer cells, hepatocytes, and liver sinusoidal endothelial cells incubated for 5 h with 2 µg/mL concentration of 10-nm SiO_2_ NPs.


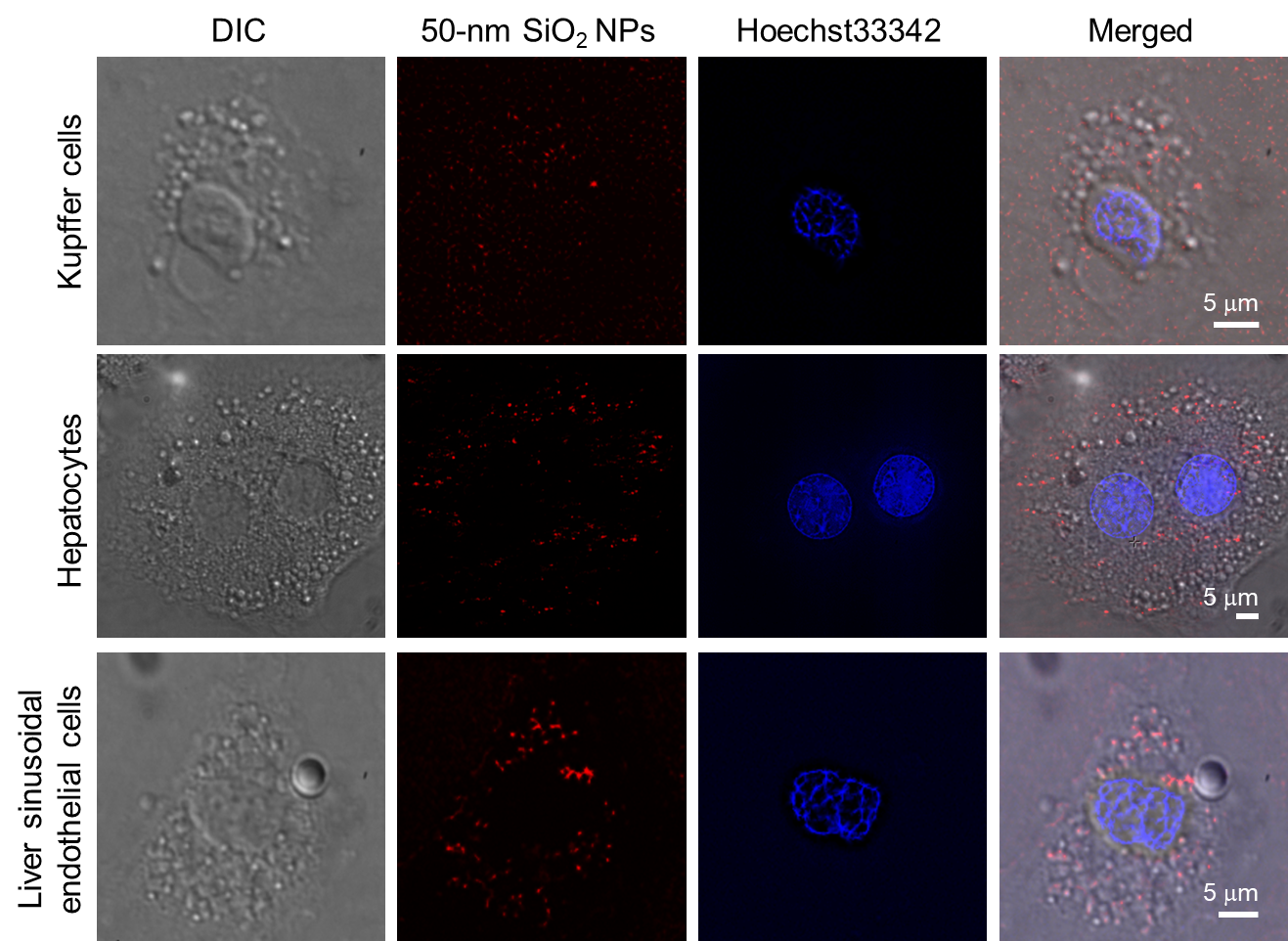


**Fig. S18.** DIC and super-resolution images of Kupffer cells, hepatocytes, and liver sinusoidal endothelial cells incubated for 5 h with 2 µg/mL concentration of 50-nm SiO_2_ NPs.


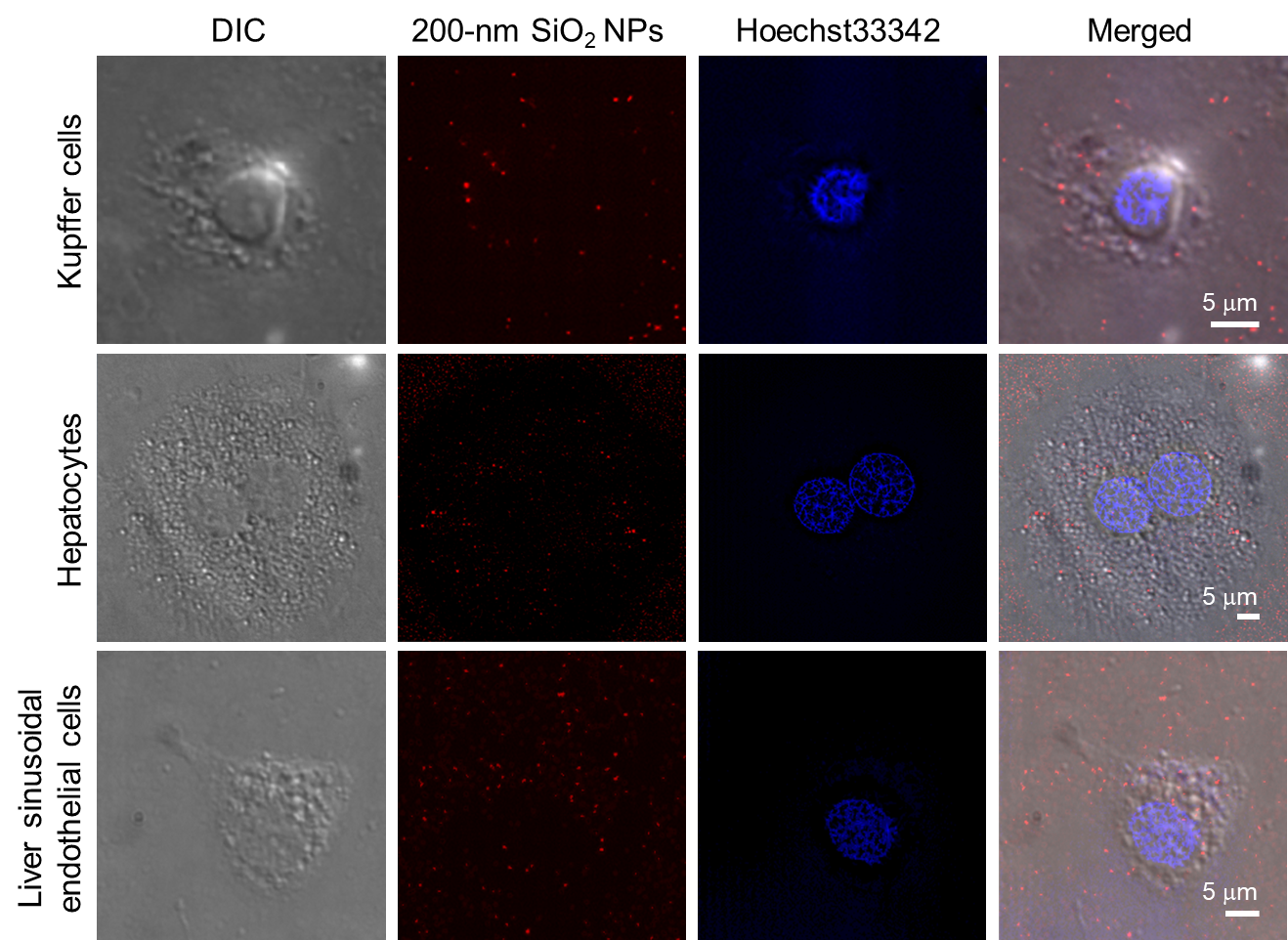


**Fig. S19.** DIC and super-resolution images of Kupffer cells, hepatocytes, and liver sinusoidal endothelial cells incubated for 5 h with 2 µg/mL concentration of 200-nm SiO_2_ NPs.


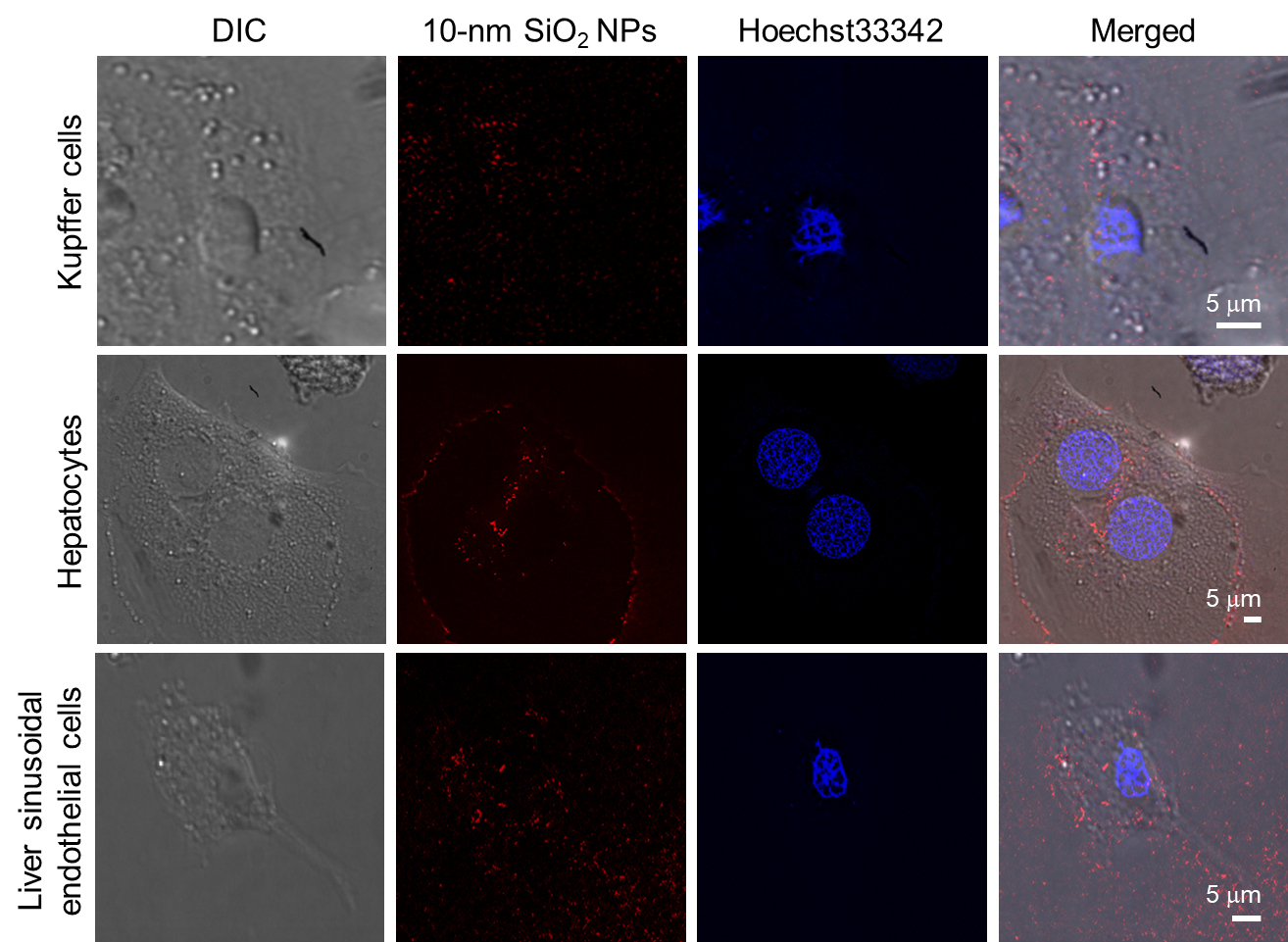


**Fig. S20.** DIC and super-resolution images of Kupffer cells, hepatocytes, and liver sinusoidal endothelial cells incubated for 24 h with 2 µg/mL concentration of 10-nm SiO_2_ NPs.


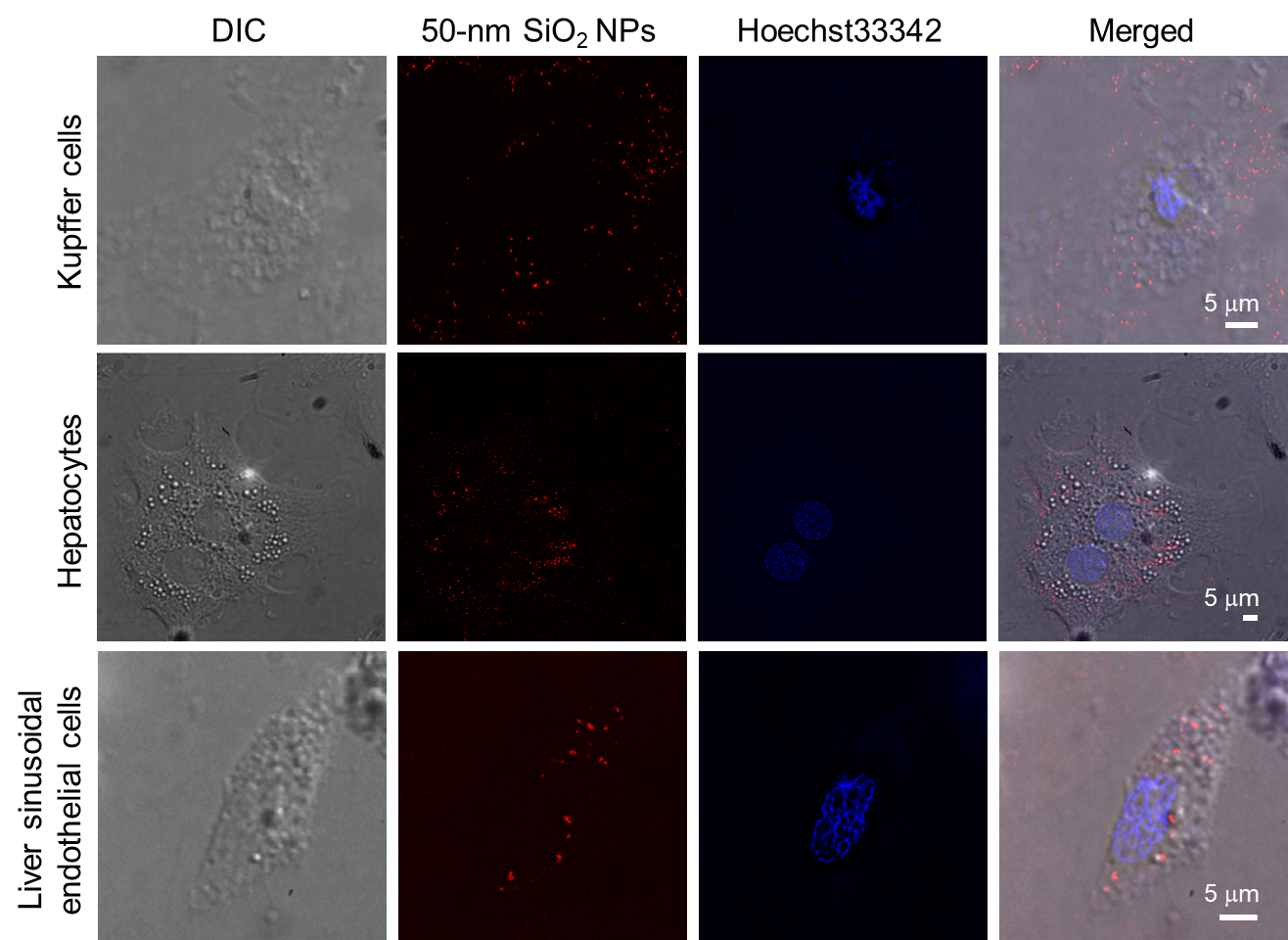


**Fig. S21.** DIC and super-resolution images of Kupffer cells, hepatocytes, and liver sinusoidal endothelial cells incubated for 24 h with 2 µg/mL concentration of 50-nm SiO_2_ NPs.


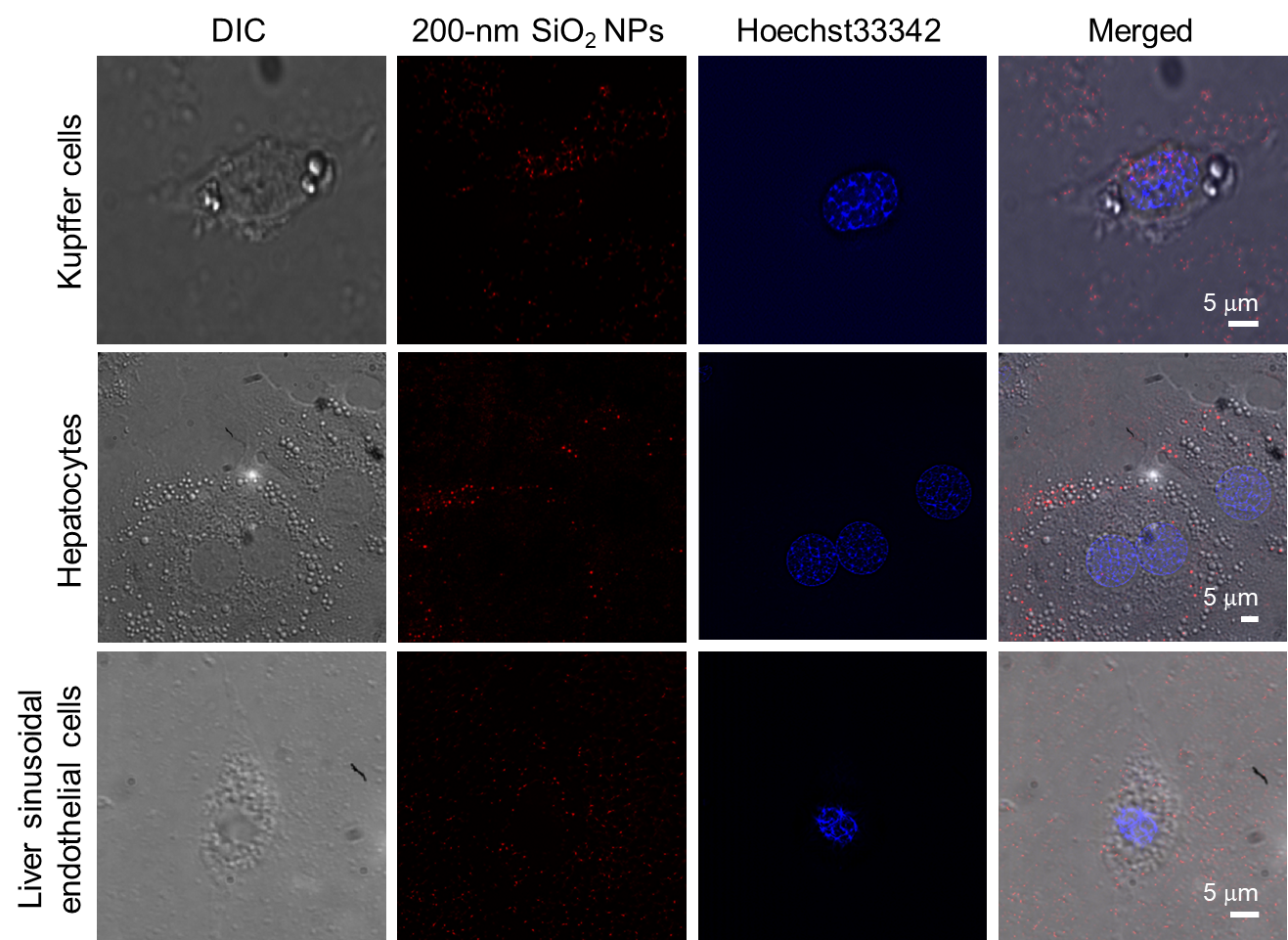


**Fig. S22.** DIC and super-resolution images of Kupffer cells, hepatocytes, and liver sinusoidal endothelial cells incubated for 24 h with 2 µg/mL concentration of 200-nm SiO_2_ NPs.

**
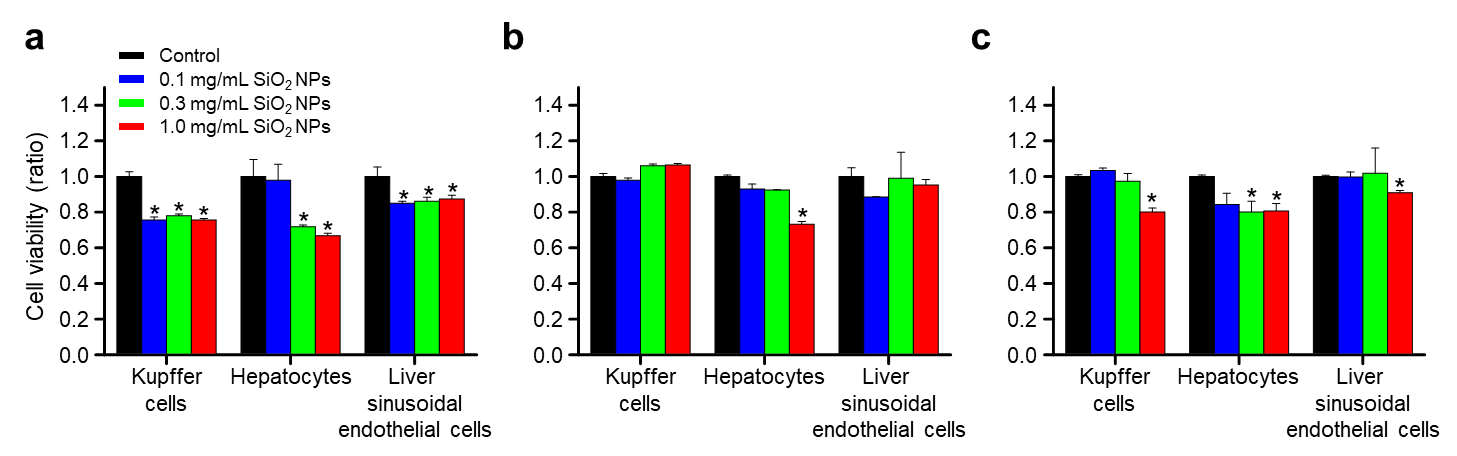
**

**Fig. S23.** Evaluation of cytotoxicity in SiO_2_ NPs-treated cells. MTS assay of **a** 10-nm, **b** 50-nm, and **c** 200-nm SiO_2_ NPs-treated Kupffer cells, hepatocytes, and liver sinusoidal endothelial cells. Cells were treated with 0.1 mg/mL, 0.3 mg/mL and 1.0 mg/mL SiO_2_ NPs for 12 h, followed by cell viability assessment using MTS assay.
